# HO_2_ + NO_2_: Kinetics, Thermochemistry, and Evidence for a Bimolecular Product Channel

**DOI:** 10.1021/acs.jpca.2c04601

**Published:** 2022-10-10

**Authors:** Kenneth McKee, Mark A. Blitz, Robin J. Shannon, Michael J. Pilling

**Affiliations:** † School of Chemistry, University of Leeds, Leeds, LS2 9JT, U.K.; ‡ National Centre for Atmospheric Science, University of Leeds, Leeds, LS2 9JT, U.K.

## Abstract

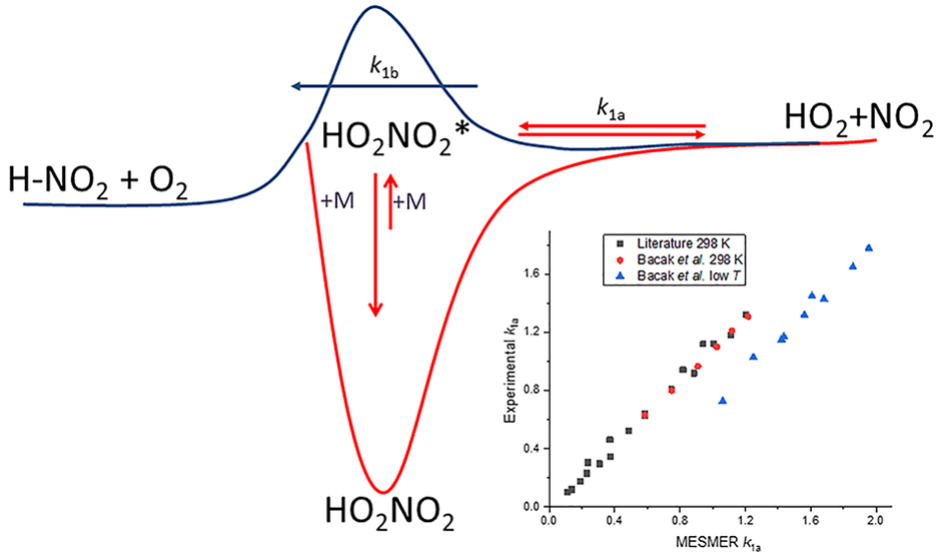

A master equation (ME) analysis of available experimental data has been carried out on the reaction HO_2_ + NO_2_ + M ⇋ HO_2_NO_2_ + M (1a)/(−1a). The analysis, based on the ME code MESMER, uses both the association and dissociation kinetic data from the literature, and provides improved thermochemistry on reaction 1a. Our preferred model assigns two low-frequency vibrations of HO_2_NO_2_ as hindered rotors and couples these to the external rotations. This model gives Δ_r_*H*°_0_(1a) = −93.9 ± 1.0 kJ mol^–1^, which implies that Δ_f_*H*°_0_ HO_2_NO_2_ = −42.0 ± 1.0 kJ mol^–1^ (uncertainties are 2σ). A significant contributor to the uncertainty derives from modeling the interaction between the internal and external rotors. Using this improved kinetics for reaction 1a/–1a, data at elevated temperatures, 353–423 K, which show no evidence of the expected equilibration, have been reanalyzed, indicating that an additional reaction is occurring that masks the equilibration. Based on a published *ab initio* study, this additional channel is assigned to the bimolecular reaction HO_2_ + NO_2_ → H–NO_2_ + O_2_ (1b); H–NO_2_ is nitryl hydride and has not previously been directly observed in experiments. The output of the master equation analysis has been parametrized and Troe expressions are provided for an improved description of *k*_1a_(*p*,*T*) and *k*_–1a_(*p*,*T*).

## 1 Introduction

Peroxy radicals are ubiquitous in the oxidation of volatile organic compounds (VOC). In combustion chemistry, they lead to branching, playing a central role in autoignition.^1^ In atmospheric chemistry, they either react with other radicals in clean environments or with NO_*x*_ in polluted environments;^2^ there is also increasing evidence that mechanisms central to autoignition have a role in atmospheric aerosol formation.^3^ The reactions of the peroxy radicals HO_2_, CH_3_O_2_, and C_4_H_9_O_2_ with NO_2_ were studied in a previous paper.^4^ At room temperature, these are association reactions that form peroxynitrates:
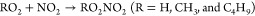
which is a chain-terminating reaction, removing peroxy radicals from the oxidation cycle. However, at higher temperatures, this reaction is not an effective sink as peroxynitrates are thermally unstable and can redissociate back to reactants. For R = CH_3_, 1,2-C_4_H_9_, the kinetics showed distinct equilibrium behavior as the temperature was increased to ∼350 K. For the reaction
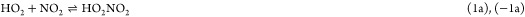
however, no distinct equilibrium behavior was observed, and no detailed data analysis was carried out.

The focus of this paper is to provide an explanation of the lack of equilibration observed in reaction 1 and to determine the equilibrium constant, *K*, and the enthalpy change of reaction. In order to do this the rate coefficients for (1a) and (−1a) need to be critically assessed, so that they can be fixed in the data analysis. Reaction 1 has been widely studied, and at 298 K, there is general consensus on the value of *k*_1_(*p*), where it is defined mainly by the determinations by Kurylo and Ouellette^5,6^ and the more recent measurements by Christensen et al.^7^ and Bacak et al.,^8^ who used more precise experimental techniques. The IUPAC recommended high- and low-pressure limits are *k*_1_^∞^ = 4.0× 10^–12^ cm^3^ molecule^–1^ s^–1^ and *k*_1_^0^ = 1.4 × 10^–31^ [N_2_] cm^3^ molecule^–1^ s^–1^; JPL recommends similar values. A problem with reaction 1 is that, at low temperatures, CH_3_OH (used to generate HO_2_) and H_2_O form complexes with HO_2_ and therefore need to be accounted for. The study by Christensen et al.^7^ made measurements on the chaperone effect of CH_3_OH in order to assign *k*_1_(*p*,*T*) over the ranges from 45 to 200 Torr and from 219 to 298 K. Using a highly sensitive chemical ionization mass spectrometer (CIMS), Bacak et al.^8^ studied the reaction with 1000 times lower radical concentrations, which in principle eliminates the interference of complexes. This study was in agreement with the literature at 298 K, but at low temperatures (223 and 200 K) their values for *k*_1_ are about a factor of 2 lower than the other measurements reported in the literature.

Master equation analysis provides an accurate description of the rate coefficients of a pressure dependent reaction, where the properties of the reactants and products are used in the calculation. The energy of the reaction is a fundamental parameter that links the forward (1a) and reverse reactions (−1a). Therefore, including the reverse kinetic data in the analysis more rigorously constrains the system parameters, particularly the energy of reaction. Zabel^9^ measured *k*_–1a_ at low temperatures (<292 K) using FTIR detection of HO_2_NO_2_, pernitric acid, and more recently, Gierczak et al.^10^ measured *k*_–1a_ between 331 and 350 K. Forward and reverse rate coefficients for pressure independent bimolecular reactions have been widely used to determine enthalpies of reaction. Pressure-dependent reactions present a greater challenge, and a master equation approach, as implemented in the present analysis, is essential.

In this paper, master equation analysis using MESMER rigorously assesses the experimental rate data for reaction 1 to provide more reliable kinetic and thermodynamic parameters and to highlight inconsistencies in the literature. Using these more reliable values for both *k*_1a_ and *k*_–1a_, our previous experimental kinetic data^4^ are reanalyzed and show that there is an additional loss channel for reaction 1; the reanalysis is facilitated by considering an *ab initio* description of the reaction 1.^11^

## 2 Master Equation Analysis

We employ a master equation analysis using the code MESMER.^12,13^ The application of the master equation to reactions in the gas phase has been extensively discussed elsewhere.^12,14−17^ Here, the main points are summarized for a reversible reaction. The energy levels of HO_2_NO_2_ are partitioned into a number of contiguous intervals or grains that are assigned values for the numbers of states they contain, average energies, and, where appropriate, average values of microcanonical rate coefficients. A single grain represents HO_2_ and pseudo first-order conditions apply ([NO_2_] ≫ [HO_2_]). These grains form the basis of the master equation representation of the system, an equation of motion of the grain probabilities, which is usually represented as
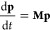
E1where **p** is a vector containing the probability densities of the grains and the matrix **M** contains the transition rates between the grains either because of collisional activation/deactivation or because of reaction. The evolution of **p** is limited by two constraints, mass (or density) conservation and detailed balance.

The solution to eq E1 can in general be written as,
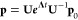
E2where **Λ** is a diagonal matrix containing the eigenvalues of **M**, **U** is a matrix of the corresponding eigenvectors, and **p**_0_ is a vector containing the initial grain densities. The number of eigenvalues is equal to the total number of grains; all the eigenvalues are negative. They can be divided into two types: internal energy relaxation eigenvalues (IEREs), which relate to the collisional relaxation of the internal energy of the system and chemically significant eigenvalues (CSEs); the magnitude of the reciprocal CSEs relate to the time scales of the chemical reactions and the number of CSEs is related to the number of chemical species included in the master equation. Generally, and certainly in the reactions analyzed here, the CSEs are significantly smaller in magnitude than the IEREs—energy relaxation occurs on time scales that are shorter than those of the chemical reaction. Under these circumstances, the rate coefficients for the chemical reactions can be determined from an analysis of the chemically significant eigenvalues and eigenvectors. In the present system, there are two CSEs, one of which, λ_1_, relates to equilibrium and is zero. The nonzero λ_2_ = −(*k*_1a_[NO_2_] + *k*_–1a_), and *K*_c_ = *k*_1a_[NO_2_]/ *k*_–1a_.

MESMER includes a facility that allows pressure- and temperature-dependent forward and reverse rate coefficients to be fitted to a reaction model. The molecular constants for the reactants and product are fed into the model and are listed in the Supporting Information. The association reaction is barrierless and the limiting high-pressure rate coefficient for association, *k*_1a_^∞^, is parametrized as *A*(*T*/298 K)^*n*^, and the microcanonical rate constants for dissociation of HO_2_NO_2_*, *k*(*E*), are determined by inverse Laplace transformation.^18^ The rates of reaction from HO_2_ + NO_2_ into a particular grain of HO_2_NO_2_ are determined by detailed balance at a specific, fixed [NO_2_].^19^ An exponential down model, coupled with detailed balance, is used for the probabilities of collisional energy transfer between the grains, based on the parametrization ⟨Δ*E*⟩_down_ = ⟨Δ*E*⟩_down,298_(*T*/298 K)^*m*^.^4^ All of the available experimental rate data were fitted to the master equation model, using the minimization of χ^2^
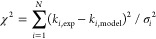
E3as the criterion of best fit. Here *k*_i,exp_ is the *i*th experimental rate coefficient, *k*_i,model_ is the model result under the same condition of *T* and *p*, *σ*_*i*_ is the standard deviation of the experimental rate coefficient, and *N* is the total number of experimental measurements. The analysis was conducted, as were the experiments, under pseudo-first-order conditions ([NO_2_] ≫ [HO_2_]), and [NO_2_] was given a fixed value of 10^15^ molecules cm^–3^). The variable parameters in the fitting process were Δ_r_*H*°_0_, *A, n*, ⟨Δ*E*⟩_down,298_ and *m*. The best available experimental data were used in a global fit for reaction 1, where all data were assigned either a 10% error or the value quoted in the paper, whichever was the greater. Rate data for both forward (association) and reverse (dissociation) data were included in the analysis, discussed in Section 3a. All of the experiments were conducted under irreversible conditions (i.e., *k*_1a_[NO_2_] ≫ *k*_–1a_ or the reverse), although experiments under equilibrating conditions are considered in Section 3b.

The vibrational and rotational constants for each species were obtained from experimental data. However, the two lowest frequency vibrations of HO_2_NO_2_ were treated as hindered rotors, HO_2_—NO_2_ and HO–ONO_2_, where the hindering potential was calculated using M062X/6-31+G**. A 1-D hindered rotor (HR) model was also used in our analysis of CH_3_ + CH_3_,^20^ and the method used is discussed in detail in our analysis of H + C_2_H_4_.^21^ However, for the present reaction, coupling between the internal and external rotors in HO_2_NO_2_ is significant. We are unable to model this interaction with a full quantum mechanical model. To overcome this problem, the MESMER analysis was carried out in four ways:

(i) The two 1-D HRs were treated quantum mechanically and uncoupled from the external rotors.
(ii) The internal and external rotors were both treated classically but coupled. This method accounts for the Coriolis interaction, the change in external moments of inertia with internal rotation, and the kinematic coupling between the internal rotors.
(iii) The HO_2_–NO_2_ rotor was treated classically and coupled to the external rotors while the HO–ONO_2_ rotor was treated quantum mechanically but uncoupled. The HO_2_–NO_2_ rotor has the greater effect on the external moments of inertia; it also has a smaller energy level spacing than the HO–ONO_2_ rotor.
(iv) Both internal rotors were treated classically but uncoupled.

The classical coupled rotors option in MESMER is based on the method discussed by Gang et al.^22^ and is described in the MESMER manual.^13^ The input parameters and methods for these calculations are given in the Supporting Information.

## 3 Results

### 3a HO_2_ + NO_2_ → HO_2_NO_2_; HO_2_NO → HO_2_ + NO_2_

The MESMER models were fitted to the literature data of Sander et al.,^23^ Kurylo et al.^5,24^ Christensen et al.^7^ and Bacak et al.^8^ for the forward reaction (1a) and of Zabel^9^ and Gierczak et al.^10^ for the reverse reaction (−1a). Their experimental rate constants are given in the Supporting Information. It is noted that the four MESMER models yielded fits of nearly equal quality to the data, as measured by χ^2^ (see Table 1). As this section only considers the fit to the experimental data, the models can be interchanged and reference to the MESMER model implies all models. The best-fit parameters for each model differ and this difference is discussed later in the thermochemistry section (section 4b).

**Table 1:** Master Equation fit to the HO_2_ + NO_2_ Data from Christensen et al.^7^ (*T* = 219–298 K), Bacak et al.^8^ (*T* = 298 K), Sander and Peterson^23^ (*T* = 298 K), and Kurylo et al.^5,24^ (*T* = 228–358 K), together with the Literature *k*_–1a_ Data from Zabel^9^ (*T* = 261–307 K) and Gierczak et al. (*T* = 331–350 K)^10^ a

parameter	model i^b^	model iii^c^	units
Δ_r_*H*°_0_	–92.6 ± 0.2	–93.9 ± 0.2	kJ mol^–1^
*A*: *k*^*∞*^ = A × (T/298)^n^	5.03 ± 1.2 × 10^–12^	5.68 ± 1.6 × 10^–12^	cm^3^ molecule^–1^ s^–1^
*n*: *k*^*∞*^ = A × (*T*/298)^*n*^	–0.20 ± 0.30	–0.11 ± 0.32	
Δ*E*_down_: Δ*E*_down,N2_ × (*T*/298)^*m*^	599 ± 76	956 ± 168	cm^–1^
*m*: Δ*E*_downN2_ × (*T*/298)^*m*^	0.25 fixed	0.25 fixed	
Δ*E*_down_: Δ*E*_down, O2_	434 ± 66	612 ± 116	cm^–1^
χ^2^/degrees of freedom	0.75	0.76	

a The low temperature data from Bacak et al. were not included in the analysis. The errors are 2σ and refer to the statistical errors derived from the fitting.

b Model i treats the internal rotors quantum mechanically but uncoupled from each other and from the external rotors.

c Model iii treats the HO–ONO_2_ rotor quantum mechanically but uncoupled and the HO_2_NO_2_ internal rotor classically and coupled to the external rotors.

**Figure 1:**
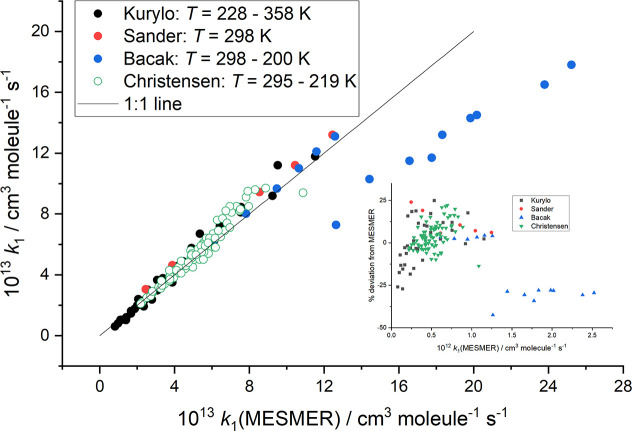
Plot of the experimental versus the MESMER calculated *k*_1a_(*p*,*T*), where the experimental data are from Christensen et al.,^7^ Bacak et al.,^8^ Sander and Peterson^23^ and Kurylo et al.^5,24^ The Bacak et al. low temperature data are incompatible with the rest of the data. The inset shows 100(*k*_i,exp_ – *k*_i,model_)/ *k*_i,model,_. Model 1 (uncoupled hindered rotors) was used in this fit.

Figure 1 shows a plot of the experimental rate coefficients versus those calculated, under identical conditions, from the best-fit MESMER model. The slope of the fit to the data is 1.00 when the Bacak et al. data are not included. The model fit to the experimental data is good except for the data of Bacak et al. at temperatures below 298 K. These data were excluded from the final analysis. From Figure 1, it can be seen that the literature values agree with those calculated within ±25% of each other. However, the low temperature (<230 K) data from Bacak et al.^8^ deviate strongly and are consistently ∼30% and up to ∼40% lower than the best fit model values; see inset Figure 1. They are clearly inconsistent with the other data. It is noted that their data at 298 K are in good agreement with the literature; see Figure 1.

Bacak et al. pointed out that in the other studies, the HO_2_ self-reaction is a significant contributor to the decay of HO_2_, (especially when chaperoned with CH_3_OH, used to generate HO_2_ in the pulsed photolysis experiments, at low temperatures) and needs to be properly accounted for to determine *k*_1a_ accurately. Bacak et al. argued that because their experiments used 1000 times lower HO_2_ radical concentrations, the HO_2_ self-reaction, and hence the uncertainty in its rate constant at low *T*, did not impact on their results. The data were reanalyzed using (i) just the data of Bacak at al., together with the dissociation data, and (ii) all the data at 298 K and above, plus the low *T* data of Bacak et al., and the dissociation data. The results are shown in the Supporting Information and demonstrate that the low *T* data of Bacak et al. are inconsistent with the Mesmer model and higher *T* association and the dissociation rate data. Both JPL^25^ and the IUPAC^26^ do not include the low temperature data from Bacak et al. in their evaluations as they noted that these data fall about a factor of 2 below the fit based on the other data. Further MESMER analysis was then performed without the 223 and 200 K data from Bacak et al.^8^ The removal of these 9 points improved the χ^2^ from 446 to 132, emphasizing that these points are incompatible with rest of the data.

**Figure 2:**
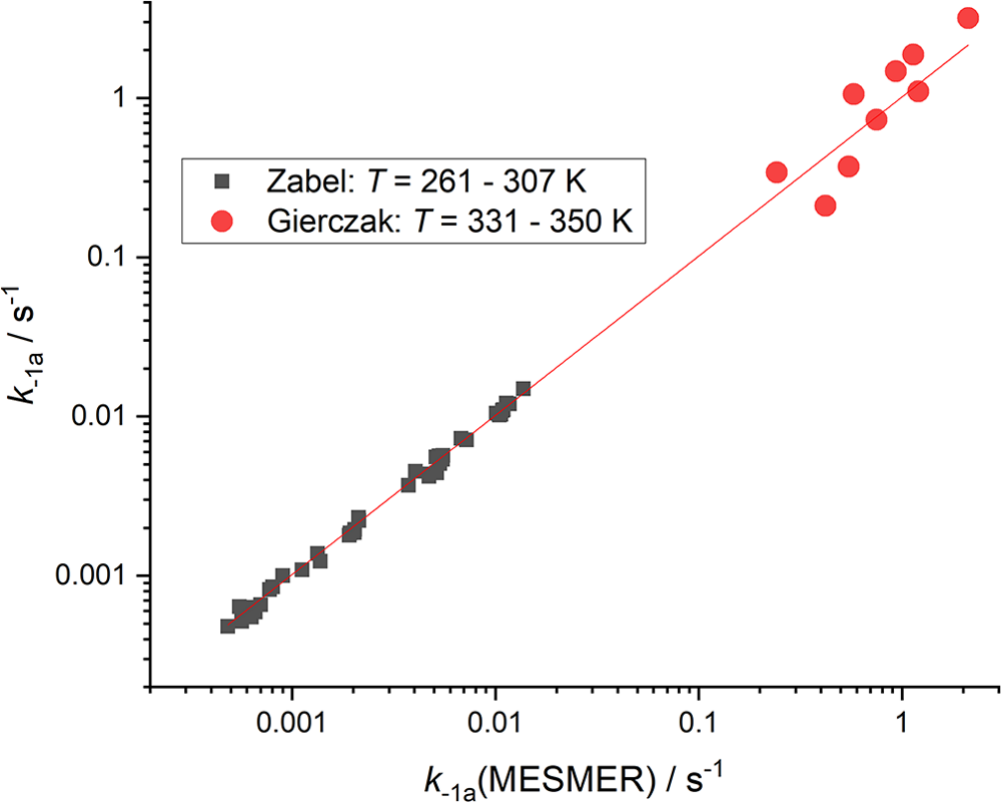
Plot of the experimental versus the MESMER calculated *k*_–1a_(*p*,*T*), where the experimental data are from Zabel^7^ and Gierczak et al.^10^ The red line has a slope equal to 1.02.

The reverse, dissociation data, *k*_–1a_, are plotted in Figure 2 against the calculated MESMER values. The linearity of the plot is very good over nearly 4 orders of magnitude in the rate constant, and the slope is close to unity. The scatter in the Zabel data (<15%) is much smaller than that in the data of Gierczak et al. (up to a factor of 2). As a result, the contribution of the dissociation reaction to the determination of the reaction enthalpy is much more strongly influenced by the Zabel data. The results of the MESMER fits to the data are given in Table 1, where the low temperature data of Bacak et al. are excluded and the error in the data of Gierczak et al. was increased to 40%.

### 3b Reanalysis of Rate Data Obtained under Equilibrating Conditions


**Figure 3:**
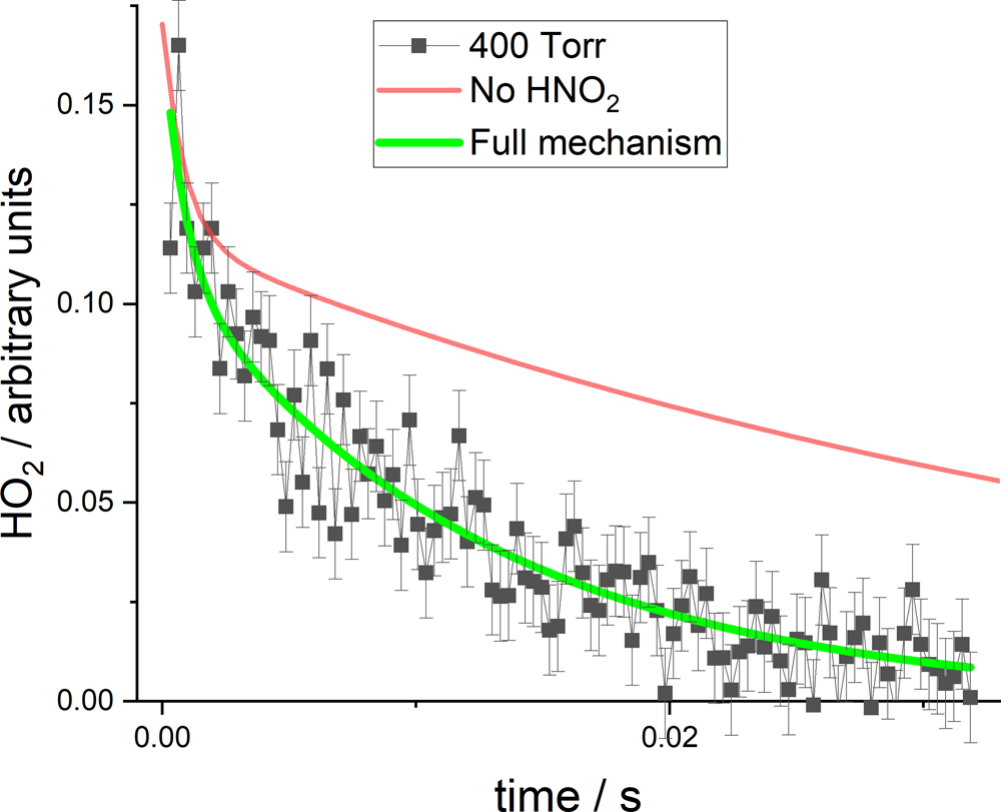
Decay trace showing the removal of HO_2_ in the presence of 38.5 mTorr NO_2_ at 423 K and a total pressure of 400 Torr. The green line is the best fit to the data and the red line is for identical conditions with reaction 1b turned off. Note that the green line starts after time-zero, but the simulation red line starts at time-zero.

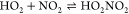
In our previous paper,^4^ we presented pulsed photolysis results for RO_2_ + NO_2_ ⇋ RO_2_NO_2_ (R = CH_3_, C_4_H_9_) under equilibrating conditions (i.e., where the dissociation and association rates are both nonzero), allowing the direct determination of the equilibrium constant and of reaction enthalpies. HO_2_ was monitored by LIF detection of the OH product, following pulsed photolysis. It was noted that attempts to fit HO_2_ + NO_2_ data under similar conditions (353–423 K) failed to return a consistent value for *k*_–1a_:
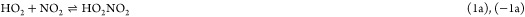


The traces did not exhibit distinctive biexponential equilibrating behavior, see Figure 3, and it was noted that, to explain the form of these traces, an additional loss process was required of either HO_2_ or HO_2_NO_2_. The possibility that HONO is a product from HO_2_ + NO_2_ had previously been explored and ruled out by Dransfield et al.^27^ and Tyndall et al.^28^ Tyndall et al. placed an upper limit of 5 × 10^–16^ cm^3^ molecule^–1^s^–1^ on the second-order rate coefficient for the contribution of this reaction to reaction 1, which is too slow to explain the observed behavior. Recently, Zhang et al.^11^ explored theoretically the bimolecular channels of reaction 1, and while the rate coefficients for the HONO channels were consistent with the literature, the channel to produce nitryl hydride:
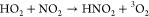
1bwas predicted to be the dominant reaction forming bimolecular products, with *k*_1b_ = 1.3 × 10^–15^ cm^3^ molecule^–1^ s^–1^. Nitryl hydride has not been observed experimentally, but it has been explored theoretically in a number of previous papers. Asatryan et al.^29^ calculated a ∼ 200 kJ mol^–1^ barrier for HNO_2_ to isomerize to HONO, where HONO is more stable. According to the Active Thermochemical Tables (ATcT)^30^ HONO is 36 kJ mol^–1^ more stable than nitryl hydride, based largely on the calculations of Klippenstein et al.^31^ This conclusion is supported by the calculations of Asatryan et al.^29^ and Zhang et al.^11^ Therefore, production of nitryl hydride from reaction 1 is a possible explanation for the attenuated equilibrium behavior seen in our high temperature data.

To explore the possibility of reaction 1b, our high temperature data were reanalyzed with reaction 1b included. The reaction scheme includes reactions 1a, –1a, and 1b, where *k*_1a_ and *k*_–1a_ are fixed to the values from our MESMER analysis, see section 3a, using either model from Table 1 to generate the *k*_1a_ and *k*_–1a_ rate coefficients at the temperatures and pressures of our high temperature experiments In the absence of NO_2_, HO_2_ is lost via self-reaction sufficiently slowly ([HO_2_] ∼ 10^13^ molecules cm^–3^) that it is reasonably approximated as a first order loss:
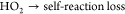
2

HO_2_ is also lost via diffusion, which is evident from the differences observed in experiments carried out at 100 and 400 Torr. Therefore, HO_2_ diffusional loss was assigned a different rate coefficient at each experimental pressure (see Supporting Information):
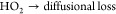
3

An additional complication is the interference from the photolysis of pernitric acid, the addition product of reaction 1a;^4^ it produces OH and interferes with the detection method used for HO_2_:
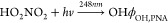
P1φ_OH,PNA_ was determined in our previous paper as 0.15 ± 0.03;^4^ the photolysis produces a ∼ 10% increase, *X*, in the baseline given by
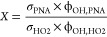
E4

As reaction 1b is an abstraction reaction with a significant barrier, *k*_1b_ was assigned an Arrhenius form, *k*_1b_ = *A*_1b_ × exp(−*E*_*a*_,_1b_/R*T*), where *A*_1b_ was fixed to 1 × 10^–10^ cm^–3^ molecule^–1^ s^–1^; the experiments were not carried out over a large enough temperature range to treat both *A*_1b_ and *E*_*a*_,_1b_ as variable parameters. The value of *E*_*a*_,_1b_ given in Table 2 is highly correlated with the assigned fixed value of *A*_1b_; i.e., the barrier for reaction 1b has a large uncertainty, but *k*_1b_ itself is well-defined over the temperature range investigated. The reaction scheme and analytical solution were given in our previous publication.^4^ It is given in an appropriately modified form in the Supporting Information, where it has been extended to include loss of HO_2_ by reaction 1b. Thirteen traces were recorded over the temperature range 343–423 K, either at 100 or 400 Torr total pressure, with helium as the bath gas. A trace at 295 K, where reaction 1b is too slow to be detected, was also included in the analysis in order to better define the photolysis of HO_2_–NO_2_, P1.

**Table 2:** Returned Parameters from Fitting the High Temperature HO_2_ + NO_2_ Data, *T* = 343–423 K (Errors Quoted Are 2σ)

parameter	value	units
*X*	0.135 ± 0.01	
*k*_1a_	fixed to MESMER fit	cm^3^ molecule^–1^ s^–1^
*k*_–1a_	fixed to MESMER fit	s^–1^
*A*_1b_	1 × 10^–10^ (fixed)	cm^3^ molecule^–1^ s^–1^
*E*_*a*__,1b_	27.4 ± 0.4	kJ mol^–1^
*k*_3_	32 ± 10	s^–1^
*k*_4_(100 Torr)	22 ± 8	s^–1^
*k*_4_(400 Torr)	4 ± 10	s^–1^
*k*_5,diff_	0.64 × *k*_4_	s^–1^
χ^2^/points	0.74	

Diffusional loss of peroxynitric acid
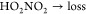
4was set equal to *k*_4_ multiplied by 0.64, the square root of the reciprocal ratio of the reagent masses. The traces were analyzed globally^32^ in order to test the mechanism and improve the parameter recovery, and the results for the variable parameters are given in Table 2. Figure 3 shows an example of the fit to the highest temperature data (*T* = 423 K). Also included in this example is a trace calculated under identical conditions with the HNO_2_ channel, reaction 1b, turned off.

Before carrying out the global analysis, each trace was fitted to the model to obtain the best fit on an individual basis. Each fit yielded a value for χ^2^ which was used for the weighting of each trace in the global analysis. In this global analysis, *k*_1a_ and *k*_–1a_ are fixed to our MESMER model—see Table 1—and the [NO_2_] is the experimental amount added for each trace. From Table 2, the overall χ^2^/point in the global analysis is 1.12, which indicates that the overall fit is not much worse than the individual fits. This indicates that the model adequately describes all the data. The rate coefficient for reaction 1b is well-defined, as evidenced by the small error returned for *E*_*a*_,_1b_. From Figure 3, it is clear that reaction 1b has a large effect on the traces and illustrates why equilibrium in the system is not readily observed. Note that the fit is constrained by the best fit values for *k*_1a_ and *k*_–1a_ determined in Section 3a. All the traces and the global fits to them are provided in the Supporting Information.

## 4 Discussion

### 4a HO_2_ + NO_2_ at Low Temperature

Our recent analysis of forward and reverse rate coefficients for H + C_2_H_4_ used electronic structure calculations of the properties of the tight transition state, floating its energy in the global fit to experimental data.^21^ The calculated and fitted transition state energies agreed well. Such an approach is not feasible for HO_2_ + NO_2_ because there is no barrier to reaction and a variational transition state method is required to calculate the microcanonical rate constants for association and dissociation. The computational costs of such an approach in global fitting to experimental data is prohibitive. Instead, we use the inverse Laplace transform (ILT) method, based on the high pressure limit for association, *k*_∞_, to determine the microcanonical rate coefficients which implicitly accounts for rotational effects.^18^ The convolution used in the ILT method is taken over all states, including rotation. The parameters for *k*_∞_, *A*, and *n* (Table 1), were floated in the global fit. The method was used in our analysis of experimental association rate coefficients for CH_3_ + CH_3_.^20^ The limiting high pressure rate coefficients returned agree well with the detailed ab initio variational calculations of Klippenstein and Harding^33^ over a wide temperature range.

The Master Equation results for *k*_1a_(*T,p*) – where *p* = N_2_ - have been parametrized using a Troe formalism^34^ and the parameters are given in Table 3. This parametrization was carried out with data over the ranges 100–500 K and 10^14^ – 10^25^ molecules cm^–3^ and the fit reproduces the Master Equation results within 7%. Also included in Table 3 are the Troe parameters from IUPAC^35^ and JPL,^36^ where it is noted that each uses a different Troe formalism. The Troe parametrization used is given in the Supporting Information.

**Table 3:** Troe Fit Parameters to MESMER-Simulated Rate Coefficients, for N_2_

	N_2_	IUPAC^26^	JPL^25^
*A*_1a_^∞^/10^–12^cm^3^ molecule^–1^ s^–1^	5.19	4.0	4.0
*n*	–0.25	0	–0.3
*A*_1a,0_/10^–31^cm^6^ molecule^–2^ s^–1^	3.87	1.4	1.9
*m*	–4.09	–3.1	–3.4
*b*	0.22	–	–
*x*_0_	0.90	–	–
*F*_centA_	0.341	0.4	0.6

**Figure 4:**
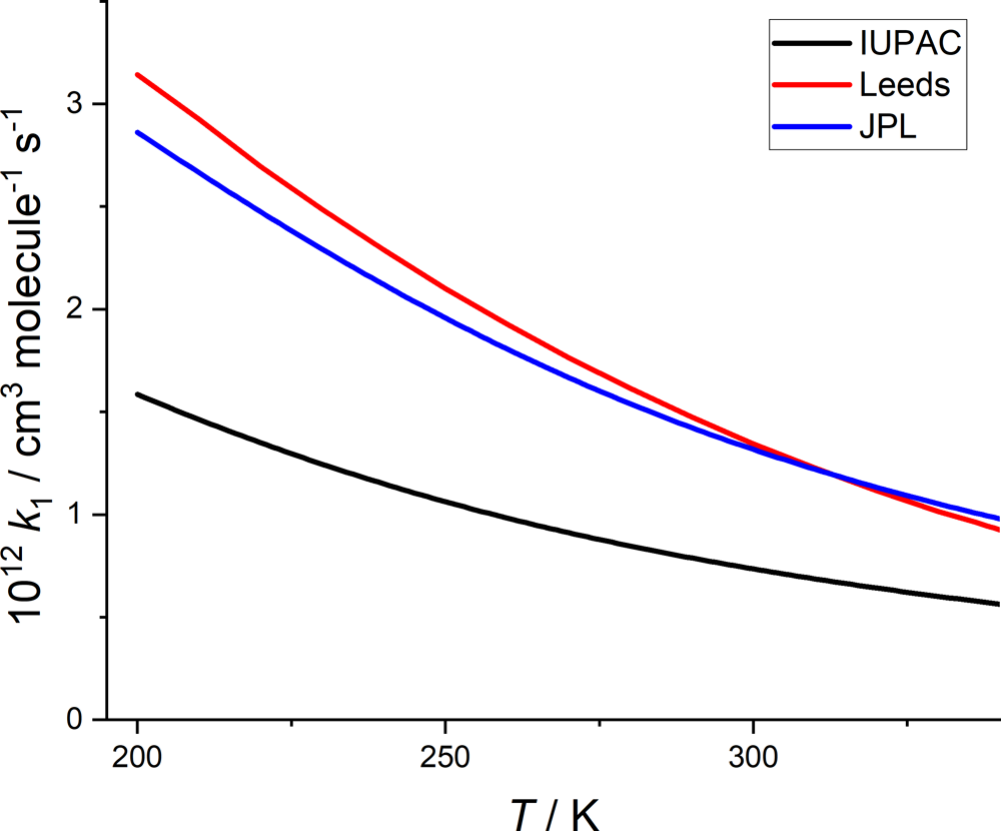
HO_2_ + NO_2_ rate coefficients predicted at 1 bar of N_2_ from the evaluations of IUPAC,^26^ JPL,^25^ and the MESMER results from this study.

Assessment of the literature data have been carried out by IUPAC^26^ and JPL,^25^ and the results at 1 bar N_2_ are given in Figure 4, together with the present results.

From this figure, it can be seen that the present results are in good agreement with JPL, especially close to 298 K. However, the IUPAC evaluation is not in agreement and is typically a factor of 2 below the other evaluations. The obvious problem with the IUPAC evaluation is that it recommends *k*_1a_(1 bar, N_2_) = 7.5 × 10^–13^ cm^3^ molecule^–1^ s^–1^ at 298 K when there is general agreement from experiments that *k*_1a_(1 bar, N_2_) is ∼1.4 × 10^–12^ cm^3^ molecule^–1^ s^–1^.^7,8^ The origin of this discrepancy is unclear. Note that none of the analyses includes the data of Bacak et al. in their recommendation.

**Figure 5:**
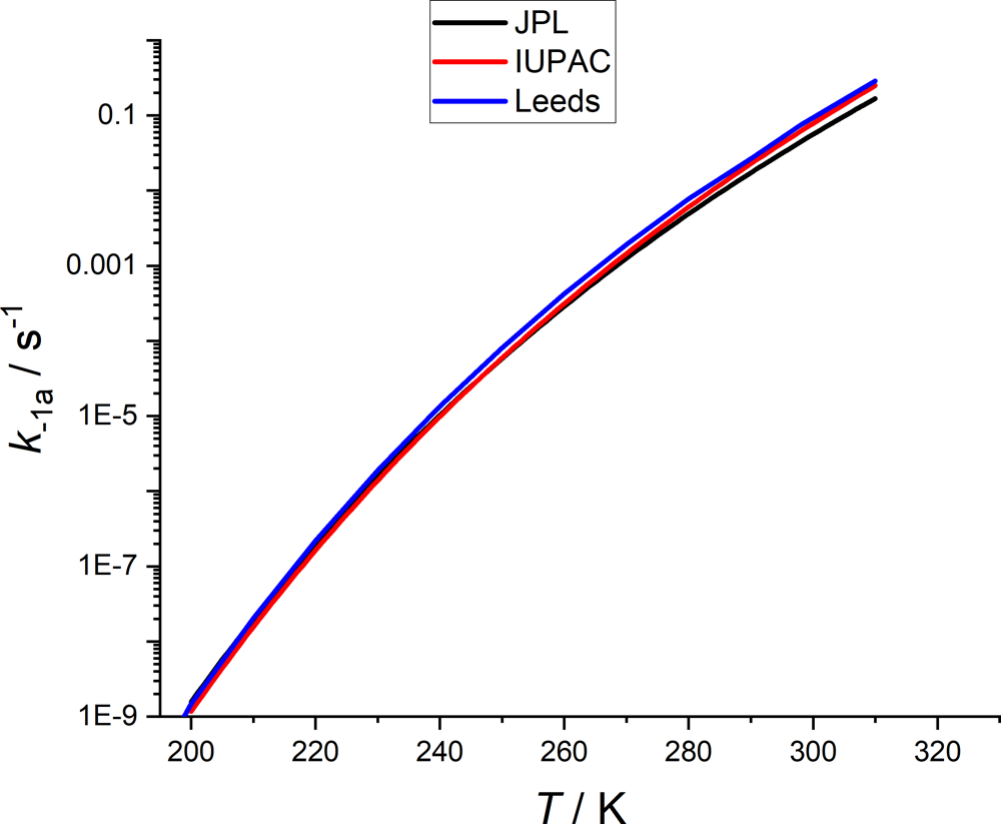
HO_2_NO_2_ decomposition rate coefficients, *k*_–1a_, predicted at 1 bar of N_2_ from the evaluations of the IUPAC, JPL, and MESMER results from this study.

In our MESMER analysis, both *k*_1a_ and *k*_–1a_ were simultaneously fitted in order to yield the most robust description of the reaction. From Figures 1 and 2, it can be seen that the MESMER model provides an excellent fit to both the forward and reverse rate coefficients and demonstrates the incompatibility of the low temperature results of Bacak et al.^8^ The IUPAC recommendation of *k*_–1a_ was based on a Troe-type fit to the literature data for *k*_–1a_ while the JPL recommendation assigned the equilibrium constant, *K*_c,1a_ with *k*_1a_(*T*,*p*) to best match the *k*_–1a_ data, i.e., *k*_–1a_ = *k*_1a_ × *K*_c,1a_. Figure 5 is a plot of *k*_–1a_(1 bar, N_2_) versus temperature from IUPAC, JPL, and Leeds, where it can be seen that there is fair agreement. In general, the Leeds assessment is closer in agreement with IUPAC, but all are within 50% of each other at all temperatures. This agreement between the assessments is also the case for *k*_–1a_^∞^.

### 4b Thermochemistry

The enthalpy and entropy of reaction 1a are given in Table 4 for this study and for previous determinations. As our analysis simultaneously considers all the forward and reverse kinetic data, it pinpoints the enthalpy of reaction with an error of less than 1 kJ mol^–1^ for each of the four models. However, it is noted that the four models predict significantly different thermodynamic parameters. The 0 K reaction enthalpies derive directly from the MESMER model and are influenced by the densities of states at all energies. The fully classical models show a large increase in the magnitude of the reaction enthalpy, but the difference between the coupled and uncoupled models is relatively small (0.6 kJ mol^–1^). This suggests that these models significantly overestimate the magnitude of the reaction enthalpy because of the use of the classical description of the internal rotors. Model iii reduces this change in the reaction enthalpy, because the HO–ONO_2_ rotor is treated quantum mechanically. The effect on the external coupling will be underestimated, but probably only slightly because the changes in the external moments of inertia resulting from this internal rotation are small. The energy level spacing in the HO_2_–NO_2_ internal rotation is significantly smaller than that of HO–ONO_2_, and so the classical description introduces a smaller error into the densities of states; by contrast, its impact on the external rotors is greater, so that inclusion of this coupling is important. Model iii therefore provides the best compromise. The use of a classical model for one of the internal rotors and the neglect of coupling for the other both introduce significant errors, so the uncertainty should be increased. A final value of −93.9 ± 1.0 kJ mol^–1^ is proposed. The resulting enthalpy of formation for HO_2_NO_2_, Δ_f_*H*°_0_, is 41.9 ± 1.0 kJ mol^–1^ based on the enthalpies of formation of HO_2_ and NO_2_ of 15.1 kJ mol^–1^ and 36.9 kJ mol^–1^ at 0 K, taken from ATcT.

**Table 4:** Thermodynamics for Reaction 1a

	Δ_r_*H*°_0_(1a)/kJ mol^–1^	Δ_r_*H°*_298_(1a)/kJ mol^–1^	Δ_r_*S*°_298_(1a)/J mol^–1^ K^–1^	Δ_f_*H*°_0_ HO_2_NO_2_/kJ mol^–1^	Δ_f_*H*°_298_ HO_2_NO_2_/kJ mol^–1^
this study: model i^a^	–92.6 ± 0.6	–96.7	–159.6	–40.6 ± 0.6	–50.5 ± 0.6
this study: model ii^b^	–95.4 ± 0.6	–99.7	–164.0	–43.4 ± 0.6	–53.4 ± 0.6
this study, model iii^c^	–93.9 ± 0.6	–98.2	–163.1	–42.0 ± 0.6	–51.9 ± 0.6
this study, model iv^d^	–94.8 ± 0.6	–99.0	–161.8	–42.8 ± 0.6	–52.7 ± 0.6
Sander et al.^23^		–96.2	–158.6		–52.7 ± 8.4
Gierczak et al.^10^		–100.4 ± 2.0	–172.0		–52.7 ± 4.2
Zabel^9^		–99.6 ± 3.0	–170.3		–51.5 ± 2.9
ATcT^38,39^	–94.6 ± 1.3	–99.3 ± 1.3	–163.9^e^	–42.6 ± 1.3	–52.9 ± 1.3
Szakacs et al.^40^	–93.3 ± 2.4	–98.7 ± 2.4	–173.0	–41.3 ± 2.4	–52.4 ± 2.4

a Model i treats the internal rotors quantum mechanically but uncoupled from each other and from the external rotors.

b Model ii treats the internal rotors classically and coupled both to each other and to the external rotors.

c Model iii treats the HO–ONO_2_ rotor quantum mechanically but uncoupled and the HO_2_NO_2_ internal rotor classically and coupled to the external rotors.

d Model iv treats both internal rotors classically but uncoupled to the external rotors.

e Available upon request from ATcT.

Table 4 also shows results for Δ_r_*H°*_298_(1a) and Δ_r_*S°*_298_(1a) from Sander et al.,^30^ Gierczak et al.,^10^ and Zabel.^9^ Sander and Petersen^23^ analyzed the equilibrium data using a second-law approach, where entropy is not fixed but determined from the temperature dependence of the equilibrium constant. This second-law approach is known to produce large errors as the equilibrium data are determined over a limited temperature range, and the values can be skewed as the entropy and enthalpy changes are highly correlated. Gierczak et al.^10^ and Zabel.^9^ used a third-law method, calculating the entropy using a vibration only model (rigid rotor harmonic oscillator, RRHO). Table 4 shows that this results in |Δ_r_*S°*_298_| > 170 J mol^–1^ K^–1^, where slight differences arise from the vibrational frequencies used, either experimental or calculated, or a combination of the two. Consequently, these two studies yield similar enthalpies of reaction; see Table 4. When the kinetic data were analyzed with MESMER using a vibration only model, the fit to the data was worse than with the HR models, with χ^2^/degrees of freedom equal to 1.59. In our recent paper on the reaction between OH and isoprene under equilibrium conditions,^37^ it was demonstrated that a vibration only model returned an incorrect value for the enthalpy of the reaction, which was better assigned when the low frequency vibrations were described as hindered rotors.

The Active Thermochemical Tables (ATcT^38,39^) are the benchmark for thermodynamic data. The major contributor to the provenance of the ATcT values for HO_2_NO_2_ is the high level electronic structure calculation of Szakacs et al.^40^ (Table 4). They calculated the hindered rotor potentials but decided to use anharmonic vibrations for *all* the modes to determine the entropy. Table 4 shows that their results for Δ_r_*S*°_298_(1a) agree with the vibration only calculations of Gierczak et al.^10^ and Zabel.^9^ Since these studies used an RRHO model, this result suggests that the effect of the anharmonicity on Δ_r_*S*°_298_ is small. Calculations for other molecules.^41−43^ also show that the effects of anharmonicity on the entropy are small at the temperatures of this study (up to 358 K). The present study shows that the influence on the entropy and enthalpy of internal rotation and its coupling with external rotation is substantial. The ATcT value for Δ_r_*S*°_298_(1a) = 163.9 J mol^–^1 K^–1^ includes anharmonicity and the coupling between the internal and external rotors. Table 4 shows that there is reasonably good agreement between our coupled rotor model iii and ATcT, both for the enthalpy and for the entropy of reaction at 298 K.

Overall, it is concluded that, to accurately assign the thermodynamics, a third-law approach beyond the vibration-only model is required. The starting point in the present system is to assign the two low-frequency vibrations as hindered rotors, where we initially considered them as two uncoupled 1-D HR. This model improved the fit to the kinetic data and returned an error (2σ) for Δ_r_*H*°_0_ of less than 0.2 kJ mol^–1^, see Table 1. However, this is purely a precision error. Alignment with ATcT is achieved when the two 1-D HR and the external rotors are coupled. This coupling of the rotors appears to be most important in accurately assigning the thermodynamics.

### 4c High Temperature Results

In the Results, reanalysis of our high temperature data revealed that a reaction model based only on equilibration is inadequate and a bimolecular product channel was added to the reaction scheme in order to provide an adequate fit to the data, see Figure 3. When these data were originally collected, it was recognized that an additional bimolecular product channel would explain the data, but the only one considered was reaction 1c:
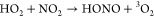
1c

However, this potential reaction had previously been investigated by Tyndall et al.^44^ and it was concluded that *k*_1c_ < 5 × 10^–16^ molecules cm^–3^ s^–1^; the rate coefficient for the bimolecular product channel at 295 K from Table 2 is equal to 1.4 × 10^–15^ molecules cm^–3^ s^–1^, which is too fast to be consistent with the results of Tyndall et al. The theoretical study on reaction 1 by Zhang et al.^11^ is more recent and provides explanation of our results as it predicts that the fastest bimolecular channel is *k*_1b_. Nitryl hydride (via reaction 1b) is a molecule that has never been observed experimentally, but has been studied theoretically. From Table 2, the value returned for the activation energy of 27.4 kJ mol^–1^ compares reasonably with the barrier height of 35 kJ mol^–1^ calculated by Zhang et al.^11^ Our HO_2_ + NO_2_ data do not cover a sufficiently extensive temperature range to determine both Arrhenius parameters, and the *A*-factor was fixed. The small error given in Table 2 does not reflect the uncertainty in *E*_*a*_,_1b_ as it is strongly correlated to the fixed value of *A*_1b_. A better comparison is via *k*_1b_; at 298 K Zhang et al.^11^ calculated 1.4 × 10^–15^ molecules cm^–3^ s^–1^ versus 1.6 × 10^–15^ molecules cm^–3^ s^–1^ from this study. While this agreement is good, there is a question mark on how Zhang et al. calculated *k*_1b_. Their *k*_1b_ decreased with increasing temperature, which is wholly inconsistent with a simple bimolecular reaction with a significant barrier.

While no experimental data exist for HNO_2_, it has been studied theoretically by Asatryan et al.,^29^ who calculated it to be 65 kJ mol^–1^ less stable than HONO-trans but there is a ∼ 200 kJ mol^–1^ barrier to this isomerization. Therefore, it can be concluded that in our high temperature experiments any HNO_2_ formed from reaction 1 will be stable. It may also be concluded that HNO_2_ does not significantly photolyze to OH, unlike HO_2_NO_2_, which was included in the data analysis scheme.

## 5 Conclusions

- The kinetics of both the forward and reverse reactions (1a1a)/(−1a), HO_2_ + NO_2_ ⇋HO_2_NO_2_, have been studied using a master equation analysis (MESMER), where the literature data were fitted simultaneously to provide the best overall parametrization of this reaction. By treating two low frequencies in HO_2_NO_2_ as hindered rotors (HR), this leads to a better fit, and four HR rotors models were considered, uncoupled and coupled with the external rotors.
- The master equation analysis provides a good fit to all the literature data except for the low temperature measurements of Bacak et al.^8^
- Previously determined high temperature HO_2_ + NO_2_ kinetic decay traces over the temperature range 353–423 K were reanalyzed where *k*_1a_(*p*,*T*) and *k*_1-a_(*p*,*T*) were fixed to the values from our MESMER analysis. This analysis revealed that the approach to equilibrium is obscured by an additional bimolecular channel, which leads to loss from the equilibrating system. Based on the literature, this channel is assigned to reaction 1b, producing nitryl hydride which has not been observed experimentally.
- The thermochemistry of reaction 1a was determined in the MESMER analysis and is compared to the literature. The four MESMER models provide equal fits to the kinetic data, but the model where the HO_2_–NO_2_ rotor was treated classically and coupled to the external rotors while the HO–ONO_2_ rotor was treated quantum mechanically but uncoupled appears to provide the best available description of the system, based on a comparison of the four models used. The use of a classical basis for the HO_2_–NO_2_ rotor undoubtedly introduces error beyond the statistical error derived from the fit to the experimental data. All the fits using the hindered rotor models are excellent, and the use of a quantum mechanical coupled model would undoubtedly provide a determination of the reaction enthalpy of very high accuracy.

## Supporting Information Available

Further MESMER analysis of the literature kinetic data, the kinetic scheme to globally analyze the high temperature HO_2_ + NO_2_ data, Troe parametrized form for *k*_–1a_(*T*,*p*) and the input file for the MESMER master equation analysis (PDF)
