# Supplementary material for: HO2 + NO2: Kinetics, Thermochemistry, and Evidence for a Bimolecular Product Channel
Source: J Phys Chem A. 2022 Oct 10;126(41):7514–22. doi: 10.1021/acs.jpca.2c04601 (PMC9589593; doi:10.1021/acs.jpca.2c04601)
Supplement: Supplementary file 1 — jp2c04601_si_001.pdf [file jp2c04601_si_001.pdf]

$HO_2 + NO_2$: Kinetics, Thermochemistry and Evidence for a Bimolecular Product Channel

Kenneth McKee,[a] Mark A. Blitz,[a,b]* Robin J. Shannon [a] and Michael J. Pilling [a]

[a] *School of Chemistry, University of Leeds, Leeds, LS2 9JT, UK*

[b] *National Centre for Atmospheric Science, University of Leeds, Leeds, LS2 9JT, UK*

**E-mail: m.blitz@leeds.ac.uk*

Supporting Information

1. Further MESMER analysis of the Bacak *et al.* data.

A MESMER fit to just the Bacak *et al.* k_{1a} data and the literature k_{-1a} data was carried out. The resulting χ^2 / point was 3.4, which is much worse than for the dataset used in Table 1. Figure SI1 shows that the MESMER fit is not able to provide a good fit to the all Bacak *et al.* data. The data fall into two distinct sets: the 298 K data and the low temperature data.

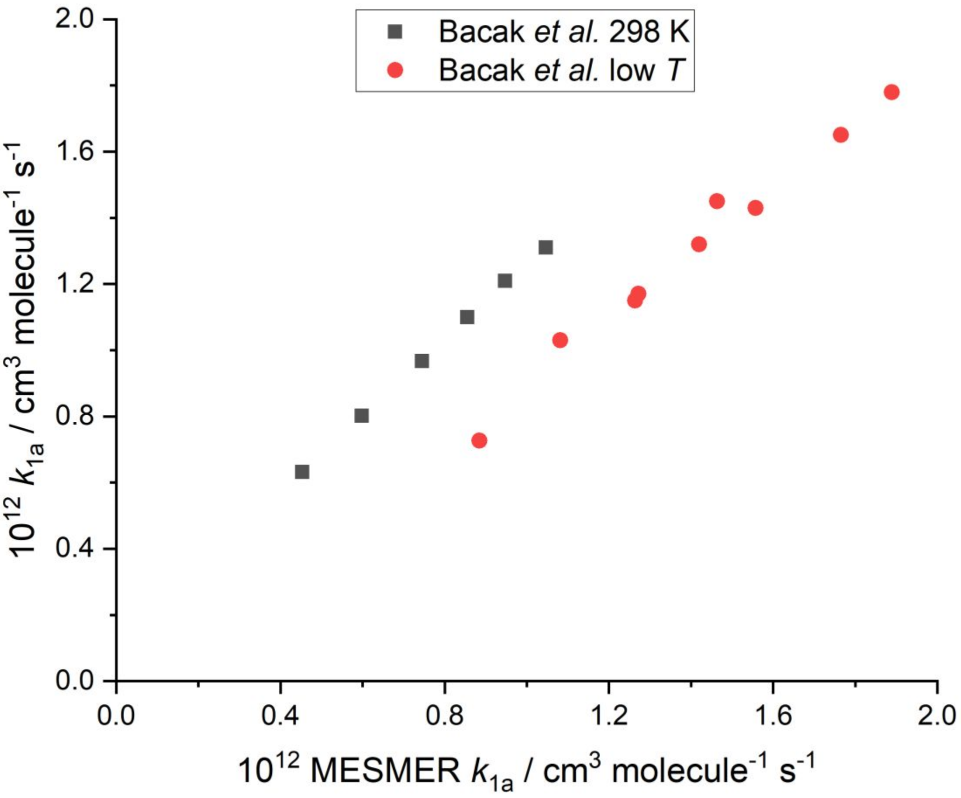

Figure SI1. Plot of the k_{1a} data from Bacak *et al.* versus the MESMER fit. The MESMER fit only considered the k_{1a} data from Bacak *et al* and the literature k_{-1a} data from Zabel [1] and Gierczak *et al.*[2]

A further MESMER fit was carried out using the literature k_{1a} data at 298 K, [3] where there is general consensus, and the low temperature data of Bacak *et al.* [4], plus the literature k_{-1a} data. The resulting χ^2 / point was 3.3, which is much worse than for the dataset used in Table 1. Figure SI2 shows the MESMER fit to the k_{1a} data, where it can be seen that all the 298 K data are in agreement and provide a good fit, but the low temperature data from Bacak *et al.* are not in agreement. Overall, it is concluded that the low temperature data from Bacak *et al.* are inconsistent with the other data and hence were not used in further MESMER analysis.

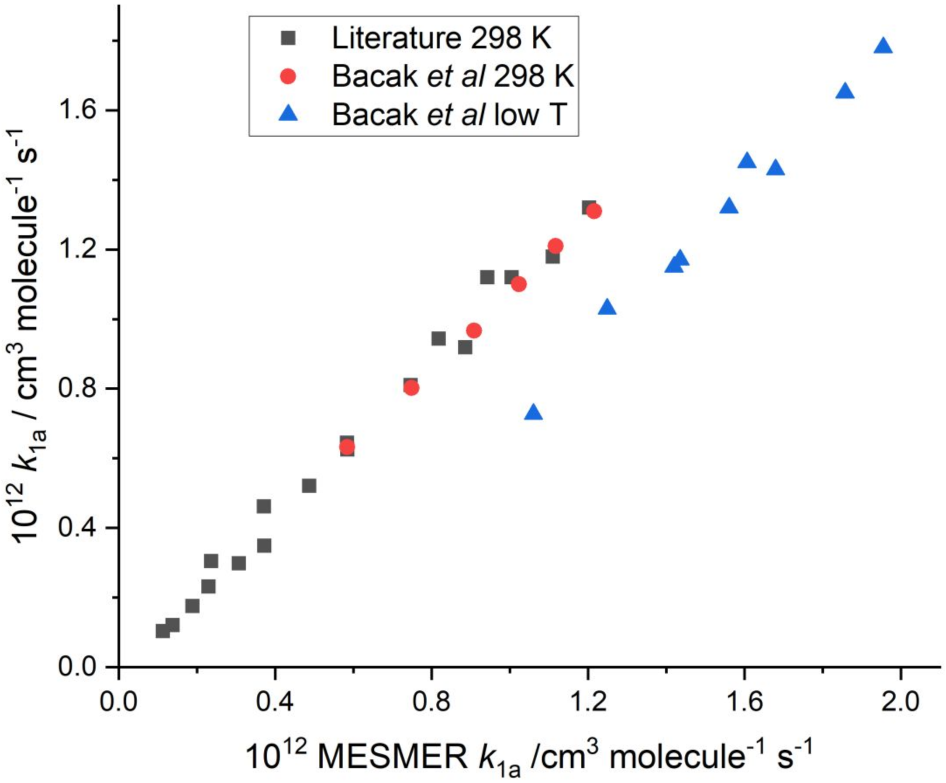

Figure SI2. Plot of the k_{1a} data from Bacak *et al.* and k_{1a} data at 298 K versus the MESMER fit. The MESMER fit used the k_{1a} data from Bacak *et al* and k_{1a} data at 298 K, plus the literature k_{-1a} data from Zabel [1] and Gierczak *et al.*[2]

2. Global analysis of kinetic traces under equilibrating conditions

A global method was used for analysing time dependent data showing the approach to equilibrium. The following kinetic scheme was used.

$HO_2 + NO_2$	➔	HO_2NO_2	$k_{1a,}$ (fixed to MESMER)
	➔	$HNO_2 + O_2$	$k_{1b} = A_{1b} \exp(-Ea_{1b}/RT)$, (labelled k_{1b} in text)
HO_2	➔	loss	k_{HO2} (labelled k_3 in text)
HO_2NO2	➔	$HO_2 + NO_2$	k_{rev1a} (labelled k_{-1a} in text, fixed to MESMER)
HO_2	➔	diffusion	k_4 (value with no added NO_2)

HO_2NO_2 ➔ diffusion k_5 (= 0.64 × k_4))

The rate constants k_4 and k_4 are diffusional loss terms for HO_2 and HO_2NO_2, respectively. The processes are relatively slow and were assumed to be a 1st order. Information onk_4 is provide via the traces taken at two pressures, 100 and 400 torr. k_5 was assigned using the relationship:

$$k_5 = [\mathrm{Mass}_{HO2}/\mathrm{Mass}_{HO2NO2}]^{0.5}\, k_4$$

HO_2NO_2 (PNA) were observed to photolyse to OH:

$$HO_2NO_2 + h\nu \xrightarrow{248nm} OH \qquad \phi_{OH,PNA}$$

This resulted in the HO_2 traces with non-zero baseline. This non-zero baseline is equal to X·[RO_2NO_2], where X is given by:

$$X = \frac{\text{cross section of PNA} \times \ \emptyset_{OH}\text{,PNA}}{\text{cross section of } HO_2 \times \emptyset_{OH}, HO_2}$$

PARAMETERS in the model = $[HO_2]_0$ (peroxy radical at time zero), PNA_{yield}, k_{1a}, k_{rev1a}, A_{1b}, Ea_{1b}, k_{HO2}, k_{4A}, k_{4B},

An ORIGIN code was used to describe the above kinetic scheme and globally fit the data

```
double A,B,C,D,F,G,L1,L2,C1,M2,M3,NO,k1,kHONO,k4,k5,PNA,k2,k3;

kHONO=(A1b*exp(-Ea1b/(8.314*TEMP)))*NO2;
k1=k1a*NO2;
k2=krev1a;
k3=0;

if (P>150){k4=k4A+kHO2+kHONO; k5=k4A*0.64;}
if (P<150){k4=k4B+kHO2+kHONO; k5=k4B*0.64;}

PNA=PNAYield;

A=-(k1+k4);
B=k2;
C=k1;
D=-(k2+k3+k5);
F=-(A+D);
G=((A*D)-(B*C));
L1=-((sqrt(F^2-4*G))+F)/2;
L2= ((sqrt(F^2-4*G))-F)/2;
C1=(A-L2)*OH/(L1-L2);

M2=(C1*exp(L1*x))+((OH-C1)*exp(L2*x));
```

M3=((C*OH)/(L1-L2))*(exp(L1*x)-exp(L2*x));
y=(M2+(M3*PNA));

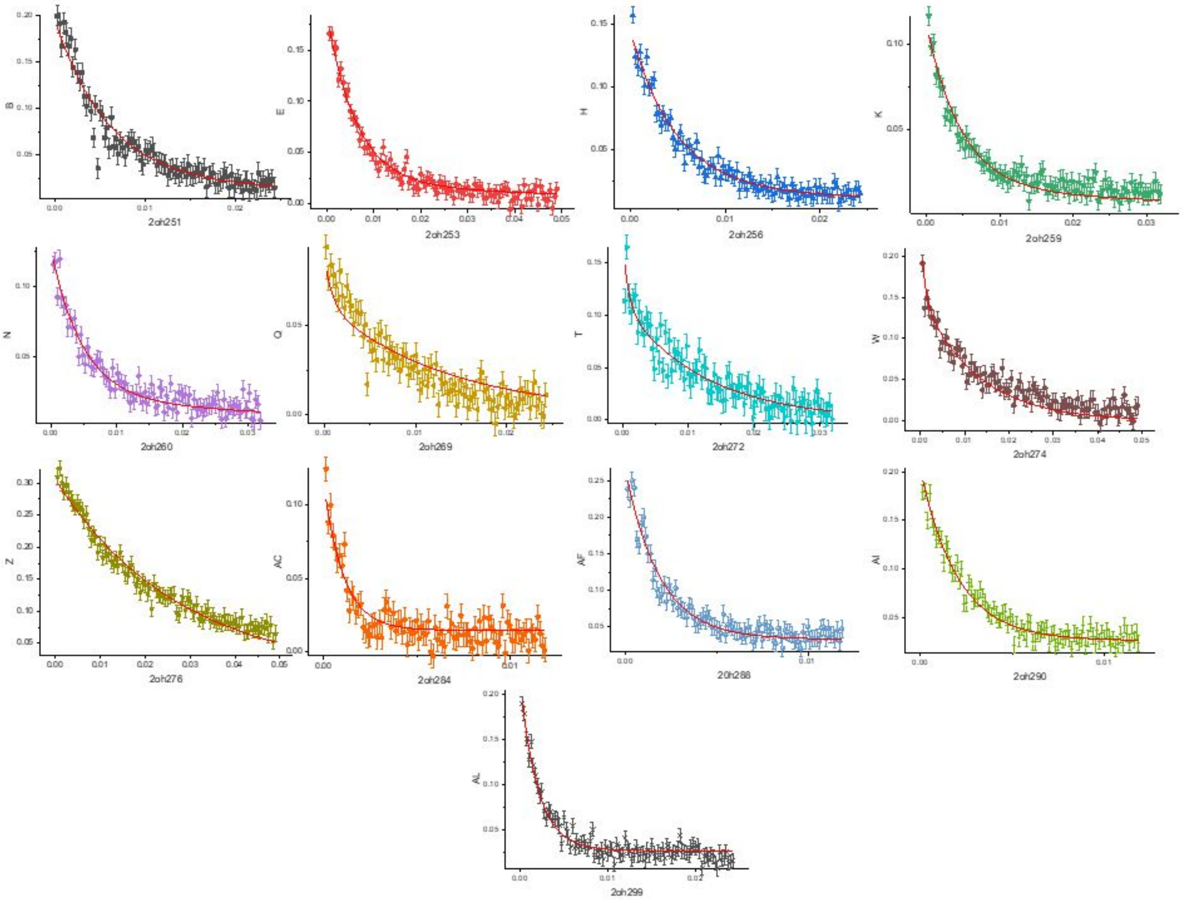

Figure SI3. Graphs of the global fit to the kinetic data. The HO_2 kinetic data were recorded over the temperature range 343 – 423 K, where the pressure (helium) was either 100 or 400 Torr and the [NO_2] was varied over the range 3.5–8.8 × 10^{14} molecule cm^{-3}; a couple of experiments had no added [NO_2] in order to define HO_2 loss. The lack of distinct equilibrium behaviour is a consequence of k_{1b}, an additional loss from the system.

3. Troe parameterisation for $k(T,p)$ for HO_2 + NO_2

$k_0(T) = A_0 \times (T/298)^n$ cm^6 molecule^{-2} s^{-1}

$k^{\infty}(T) = A^{\infty} \times (T/298)^m$ cm^3 molecule^{-1} s^{-1}

Broadening parameters based on Troe and Ushakov [5]

For $x = k_0[\mathrm{M}]/k_{\infty}$,

Troe and Ushakov give $F(x) = (1 + \frac{x}{x_0})/[1 + (\frac{x}{x_0})^n]^{1/n}$

with $n = \left[\frac{\ln 2}{\ln\left(\frac{2}{F_{\text{cent}}}\right)}\right][1 - b + b\left(\frac{x}{x_0}\right)^q]$

where $q = (F_{\text{cent}} - 1)/\ln(\frac{F_{\text{cent}}}{10})$, the parameter x_0 is in the range 0.9 – 1.1 and b is in the range 0.1 – 0.25.

Parameterized form of $F_{\text{cent}}(T) = F_{\text{cent}}\text{A} \times \exp(-F_{\text{cent}}\text{B} \times T) + F_{\text{cent}}\text{C}$

The Troe parameters for $k_{1a}(T,p)$ are given in Table 3 in the main text. The $k_{-1a}(T,p)$ Master Equation data has been parametrized using the same formalism except that the limiting rate coefficients has an extra term for the barrier, E_a:

$k_0(T) = A_0 \times (T/298)^n \times \exp(-E_{a\,0}/(8.314 \times T))$ cm^3 molecule^{-1} s^{-1}

$k^{\infty}(T) = A^{\infty} \times (T/298)^m \times \exp(-E_a^{\infty}/(8.314 \times T))$ s^{-1}

Table SI1. Troe fit parameters to MESMER-simulated rate coefficients, for N_2.

	N_2	IUPAC
A_{-1a}^{∞}/ 10^{16} s^{-1}	1.39	0.6
n	-0.65	0
$E_{a\ -1a}^{\ \infty}$ / kJ mol^{-1}	95170	92870
$A_{-1a,0}$ / 10^{-31} cm^3 $molecule^{-1}$ s^{-1}	0.0151	4.1×10^{-5}
m	-4.97	0
$E_{a\ -1a}^{\ 0}$ / kJ mol^{-1}	96020	88540
b	0.22	-
x_0	0.9	-
F_{centA}	0.343	0.4
k_{-1a} (1 bar N_2) / s^{-1}	0.074	0.062

4. Input file for MESMER master equation analysis

HO_2 + NO_2 Mesmer xml input


```
<me:title> HO2 NO2 association</me:title>
  <moleculeList>


<molecule xmlns="http://www.xml-cml.org/schema" id="HOONO2">
 <atomArray>
  <atom id="a1" elementType="N" x3="0.622400" y3="0.081500" z3="-0.000200"/>
  <atom id="a2" elementType="O" x3="-0.604600" y3="-0.803700" z3="0.067500"/>
  <atom id="a3" elementType="O" x3="-1.751600" y3="0.017400" z3="-0.146200"/>
  <atom id="a4" elementType="O" x3="1.616800" y3="-0.599200" z3="-0.023000"/>
  <atom id="a5" elementType="O" x3="0.435900" y3="1.270300" z3="0.007800"/>
  <atom id="a6" elementType="H" x3="-1.929100" y3="0.351200" z3="0.752700"/>
 </atomArray>
 <bondArray>
  <bond id="b1" atomRefs2="a1 a2" order="1"/>
  <bond id="b2" atomRefs2="a1 a4" order="2"/>
  <bond id="b3" atomRefs2="a1 a5" order="2"/>
  <bond id="b4" atomRefs2="a2 a3" order="1"/>
  <bond id="b5" atomRefs2="a3 a6" order="1"/>
 </bondArray>

                  <propertyList>
       <property dictRef="me:ZPE">
         <!--scalar units="kJ/mol">-42.8</scalar-->
  <scalar units="kJ/mol" lower="-70" upper="-20.0" stepsize="0.1">-40.47</scalar>

       </property>
       <property dictRef="me:rotConsts">
         <array units="cm-1">0.40009              0.15562     0.11332</array>
       </property>
       <property dictRef="me:symmetryNumber">
         <scalar>1</scalar>
       </property>


       <property dictRef="me:frequenciesScaleFactor">
       <!-- <scalar>0.9</scalar> -->
       <scalar>0.916</scalar>
       </property>
       <!-- <property dictRef="me:vibFreqs"> -->
       <!-- Ravi -->
       <!-- <array units="cm-1">3540 1728 1397 1304 945 803 722 654 483 340 310 145</array> -->
       <!-- </property> -->

             <!-- Hessian vibs 159.303 330.51 396.624 558.493 732.164 800.827 878.538 1086.47 1404.25 1472.5  -->
             <!-- 1832.19 3778.98  -->

<property title="Hessian" dictRef="me:hessian">
<matrix rows="18" matrixType="squareSymmetricLT" units="Hartree/Bohr2" >
7.42572997E-01 -3.39900850E-01 1.03008013E+00 -6.24001398E-03  4.85817791E-04
  2.59865157E-01 -4.47092430E-02 -6.97733154E-03 -2.44426775E-03  3.45414964E-01
  3.02898406E-02 -1.29913903E-01  1.41415962E-02 -1.13152322E-01  3.60140627E-01
 -6.39257815E-04  8.64517794E-03 -6.33564184E-02  1.73651508E-02 -2.50216689E-02
  6.03456165E-02 -5.39543536E-02  1.65099375E-02 -9.18898092E-03 -1.84907793E-01
  9.12466550E-02 -4.81125388E-02  2.97146759E-01 -2.29633813E-03  2.92548299E-03
 -1.20820013E-02  1.46965411E-01 -1.54917765E-01  3.47744305E-02 -1.57066964E-01
  2.20925157E-01 -6.19869637E-03 -1.04058224E-02 -3.58271586E-03 -7.56428988E-03
  7.10514945E-04 -5.80776690E-02 -1.92505182E-02  1.59291742E-01  4.94607069E-01
 -4.92488347E-01  2.35141211E-01  8.61508464E-03 -8.23106028E-02  1.55967098E-02
  1.49211495E-03 -1.74346080E-02 -3.65688881E-03  4.12681583E-03  5.99398300E-01
  2.40884724E-01 -2.89401953E-01 -3.59862756E-03 -7.10831771E-03  2.75290592E-02
 -1.58582020E-03 -2.76793610E-03  3.52338618E-03  1.69032057E-03 -3.17089051E-01
  3.46493480E-01  7.15393894E-03 -4.43201336E-03 -9.65858433E-02  4.73214796E-03
 -3.21506569E-03  1.77772571E-02  1.48523456E-03  3.01355203E-03  5.47127953E-03
 -1.21681991E-02  6.37067337E-03  3.92201994E-02 -1.48244413E-01  8.34071995E-02
  1.69065387E-03 -6.77156698E-03 -4.86252118E-02 -5.11758323E-04 -6.81677971E-04
```

-1.66861394E-02 1.05680625E-03 -6.77545950E-03 8.86043089E-02 6.21303459E-04
1.61078911E-01 6.98618499E-02 -6.22819164E-01 -1.57621366E-03 -3.20602977E-02
-8.13242926E-02 4.01406273E-03 2.42945190E-03 1.11769121E-02 2.14560477E-03
7.14966718E-02 -8.69402064E-02 -1.53702762E-03 -1.07541100E-01 7.83401946E-01
1.74435778E-03 2.85364173E-03 -9.56225233E-02 3.55273805E-05 2.54447482E-03
2.52928665E-02 4.68516166E-04 4.89015386E-04 -3.49661820E-04 1.31803530E-03
-3.07076524E-03 3.42408347E-02 -1.83465146E-03 -1.51714650E-03 3.66730449E-02
-3.17664020E-03 1.18198337E-02 7.56752414E-03 -2.67157583E-02 2.46443283E-02
3.04062892E-02 -4.01683262E-02 3.27409196E-02 2.78298824E-02 -3.89282237E-04
-2.52372847E-03 -1.82442580E-03 1.39420576E-03 -4.18657608E-03 -1.73178516E-03
6.90558012E-02 1.16077340E-03 9.12941100E-03 2.62942854E-03 1.23328581E-02
-2.15137260E-02 -2.08261820E-02 4.96488559E-02 -8.36331732E-02 -1.53432359E-01
-1.48865278E-03 -1.20376577E-03 -2.00118728E-04 8.40942559E-04 -3.49519547E-03
-1.29922020E-03 -6.24947771E-02 1.00716449E-01 4.17967146E-03 2.85319827E-03
-7.17656286E-04 -1.21242685E-02 1.08401486E-02 1.80183473E-02 7.45982871E-02
-1.85486738E-01 -4.38068302E-01 -3.38385161E-03 1.94219067E-04 -1.23727410E-04
-1.02235379E-03 -1.52927971E-03 -2.34560895E-04 -6.22474847E-02 1.73128452E-01
4.21125900E-01> </matrix>
</property>

<property dictRef="me:MW">
<scalar units="amu">79</scalar>
</property>
<property dictRef="me:epsilon">
<scalar>380</scalar>
</property>
<property dictRef="me:sigma">
<scalar>4.93</scalar>
</property>

<property dictRef="me:spinMultiplicity">
<scalar>1</scalar>
</property>
</propertyList>

<me:DOSCMethod>QMRotors</me:DOSCMethod>

<me:ExtraDOSCMethod xsi:type="HinderedRotorQM1D">
<me:bondRef>b1</me:bondRef>
<me:HinderedRotorPotential format="numerical" units="kJ/mol" expansionSize="11" UseSineTerms="yes" scale="1">
<me:PotentialPoint angle=" 0” potential=" 0”/>
<me:PotentialPoint angle=" 15” potential=" 1.62781”/>
<me:PotentialPoint angle=" 30” potential=" 7.535185”/>
<me:PotentialPoint angle=" 45” potential=" 16.199335”/>
<me:PotentialPoint angle=" 60” potential=" 25.30982”/>
<me:PotentialPoint angle=" 75” potential=" 31.847315”/>
<me:PotentialPoint angle=" 90” potential=" 33.6064”/>
<me:PotentialPoint angle=" 105” potential=" 30.35078”/>
<me:PotentialPoint angle=" 120” potential=" 23.708265”/>
<me:PotentialPoint angle=" 135” potential=" 15.201645”/>
<me:PotentialPoint angle=" 150” potential=" 7.45642”/>
<me:PotentialPoint angle=" 165” potential=" 2.231675”/>
<me:PotentialPoint angle=" 180” potential=" -0.026255”/>
<me:PotentialPoint angle=" 195” potential=" 2.336695”/>
<me:PotentialPoint angle=" 210” potential=" 8.900445”/>
<me:PotentialPoint angle=" 225” potential=" 17.53834”/>
<me:PotentialPoint angle=" 240” potential=" 26.018705”/>
<me:PotentialPoint angle=" 255” potential=" 32.08361”/>
<me:PotentialPoint angle=" 270” potential=" 33.527635”/>
<me:PotentialPoint angle=" 285” potential=" 29.66815”/>
<me:PotentialPoint angle=" 300” potential=" 22.36926”/>
<me:PotentialPoint angle=" 315” potential=" 13.91515”/>
<me:PotentialPoint angle=" 330” potential=" 6.747535”/>
<me:PotentialPoint angle=" 345” potential=" 2.179165”/>
<me:PotentialPoint angle=" 360” potential=" 0”/>
</me:HinderedRotorPotential>
<me:periodicity>2</me:periodicity>
<me:PlotStates/>
</me:ExtraDOSCMethod>

```
<me:ExtraDOSCMethod xsi:type="HinderedRotorQM1D">
<me:bondRef>b4</me:bondRef>
<me:HinderedRotorPotential format="numerical" units="kJ/mol" expansionSize="11" UseSineTerms="yes"  scale="1">
        <me:PotentialPoint  angle="    0” potential="      0”/>
        <me:PotentialPoint  angle="    15” potential="     0.57761”/>
        <me:PotentialPoint  angle="    30” potential="     2.36295”/>
        <me:PotentialPoint  angle="    45” potential="     4.699645”/>
        <me:PotentialPoint  angle="    60” potential="     6.93132”/>
        <me:PotentialPoint  angle="    75” potential="     8.4016”/>
        <me:PotentialPoint  angle="    90” potential="     8.637895”/>
        <me:PotentialPoint  angle="    105” potential="    7.640205”/>
        <me:PotentialPoint  angle="    120” potential="    5.61857”/>
        <me:PotentialPoint  angle="    135” potential="    3.229365”/>
        <me:PotentialPoint  angle="    150” potential="    1.15522”/>
        <me:PotentialPoint  angle="    165” potential="    0.078765”/>
        <me:PotentialPoint  angle="    180” potential="    0.42008”/>
        <me:PotentialPoint  angle="    195” potential="    2.546735”/>
        <me:PotentialPoint  angle="    210” potential="    6.222435”/>
        <me:PotentialPoint  angle="    225” potential="    10.39698”/>
        <me:PotentialPoint  angle="    240” potential="    13.836385”/>
        <me:PotentialPoint  angle="    255” potential="    15.884275”/>
        <me:PotentialPoint  angle="    270” potential="    16.22559”/>
        <me:PotentialPoint  angle="    285” potential="    14.80782”/>
        <me:PotentialPoint  angle="    300” potential="    11.86726”/>
        <me:PotentialPoint  angle="    315” potential="    7.850245”/>
        <me:PotentialPoint  angle="    330” potential="    3.806975”/>
        <me:PotentialPoint  angle="    345” potential="    1.023945”/>
        <me:PotentialPoint  angle="    360” potential="    0”/>
</me:HinderedRotorPotential>
    <me:PlotStates/>
        </me:ExtraDOSCMethod>


<!-- <me:DOSCMethod xsi:type="me:ClassicalCoupledRotors"> -->
  <!-- <me:MCPoints>10000</me:MCPoints> -->
 <!-- <me:RotorArray> -->
<!-- <me:Rotor bondRef="b1" periodicity="2"> -->
<!-- <me:HinderedRotorPotential format="numerical" units="kJ/mol" expansionSize="11" UseSineTerms="yes"  scale="1"> -->
XXXXXXXXXXXXXXXXXXXXXXXXXXXXXXXXXXXXXXXXXXXXXXXXXX

<!-- </me:HinderedRotorPotential> -->
        <!-- <me:periodicity>2</me:periodicity> -->
  <!-- </me:Rotor> -->

<!-- <me:Rotor bondRef="b4" periodicity="1"> -->
<!-- <me:HinderedRotorPotential format="numerical" units="kJ/mol" expansionSize="11" UseSineTerms="yes"  scale="1"> -->
XXXXXXXXXXXXXXXXXXXXXXXXXXXXXXXXXXXXXXXXXXXXXXXXXXXXXXXXXXXXXXX

 <!-- </me:HinderedRotorPotential> -->
<!-- </me:Rotor> -->
<!-- </me:RotorArray> -->
<!-- </me:DOSCMethod>        -->


<me:ExtraDOSCMethod xsi:type="HinderedRotorCM1D">
  <me:bondRef>b1</me:bondRef>
   <me:HinderedRotorPotential format="numerical" units="kJ/mol" expansionSize="11" UseSineTerms="yes"  scale="1">
       XXXXXXXXXXXXXXX
</me:HinderedRotorPotential>
       <me:periodicity>2</me:periodicity>
       <me:PlotStates/>
  </me:ExtraDOSCMethod>


<me:ExtraDOSCMethod xsi:type="HinderedRotorCM1D">
<me:bondRef>b4</me:bondRef>
<me:HinderedRotorPotential format="numerical" units="kJ/mol" expansionSize="11" UseSineTerms="yes"  scale="1">
```

```
XXXXXXXXXXXXXXXXXXXXXXXXXXXXXXXXXXXXXX
</me:HinderedRotorPotential>
<me:PlotStates/>
</me:ExtraDOSCMethod>


<me:energyTransferModel xsi:type="me:ExponentialDown">
<me:deltaEDown bathGas="N2" units="cm-1" lower="125" upper="2000" stepsize="10">597</me:deltaEDown>
  <me:deltaEDownTExponent bathGas="N2" referenceTemperature="298" >0.25</me:deltaEDownTExponent>

<!-- <me:deltaEDown bathGas="He" units="cm-1" lower="125" upper="600" stepsize="10">200</me:deltaEDown> -->
 <!-- <me:deltaEDownTExponent bathGas="He" referenceTemperature="298" >1.0</me:deltaEDownTExponent> -->

<me:deltaEDown bathGas="O2" units="cm-1" lower="125" upper="2000" stepsize="10">377</me:deltaEDown>
<me:deltaEDownTExponent bathGas="O2" referenceTemperature="298" >0.25</me:deltaEDownTExponent>

</me:energyTransferModel>

</molecule>

<molecule xmlns="http://www.xml-cml.org/schema" id="NO2">
 <atomArray>
  <atom id="a1" elementType="N" x2="0.000000" y2="0.000000"/>
  <atom id="a2" elementType="O" x2="0.000000" y2="1.104700"/>
  <atom id="a3" elementType="O" x2="0.000000" y2="-1.104700"/>
 </atomArray>
 <bondArray>
  <bond atomRefs2="a1 a2" order="1"/>
  <bond atomRefs2="a1 a3" order="1"/>
 </bondArray>
  <propertyList>
    <property dictRef="me:ZPE">
     <scalar units="kJ/mol">36.86</scalar>
    </property>
    <property dictRef="me:rotConsts">
     <array units="cm-1">8.00120  0.433640.41040
                </array>
    </property>
    <property dictRef="me:symmetryNumber">
     <scalar>2</scalar>
    </property>
    <property dictRef="me:frequenciesScaleFactor">
     <scalar>1</scalar>
    </property>
    <property dictRef="me:vibFreqs">
     <array units="cm-1">1318  750      1618</array>
    </property>
    <property dictRef="me:MW">
     <scalar units="amu">46</scalar>
    </property>
        <property dictRef="me:epsilon">
     <scalar>146</scalar>
    </property>
    <property dictRef="me:sigma">
     <scalar>4.68</scalar>
    </property>
    <property dictRef="me:spinMultiplicity">
     <scalar>2</scalar>
    </property>
   </propertyList>
        <me:DOSCMethod>QMRotors</me:DOSCMethod>

   <me:energyTransferModel xsi:type="me:ExponentialDown">
        <me:deltaEDown bathGas="N2" units="cm-1" lower="125" upper="2600" stepsize="10">1597</me:deltaEDown>
          <me:deltaEDownTExponent bathGas="N2" referenceTemperature="298" >0.25</me:deltaEDownTExponent>

       <me:deltaEDown bathGas="O2" units="cm-1" lower="125" upper="2600" stepsize="10">977</me:deltaEDown>
          <me:deltaEDownTExponent bathGas="O2" referenceTemperature="298" >0.25</me:deltaEDownTExponent>

   </me:energyTransferModel>
</molecule>
```

```
  <molecule id="HO2">
   <atomArray>
     <atom id="a1" elementType="H" />
     <atom id="a2" elementType="O" />
     <atom id="a3" elementType="O" />
   </atomArray>
   <bondArray>
     <bond atomRefs2="a1 a2" order="1" />
     <bond atomRefs2="a2 a3" order="1" />
   </bondArray>
   <propertyList>
     <property dictRef="me:ZPE">
      <scalar units="kJ/mol">15.12</scalar>
     </property>
     <property dictRef="me:rotConsts">
      <array units="cm-1">20.35652	1.11803	1.05632  </array>
     </property>
     <property dictRef="me:symmetryNumber">
      <scalar>1</scalar>
     </property>
     <property dictRef="me:frequenciesScaleFactor">
      <scalar>1.0</scalar>
     </property>
     <property dictRef="me:vibFreqs">
      <array units="cm-1">3436  1392	1098			</array>
     </property>
     <property dictRef="me:MW">
      <scalar units="amu">33</scalar>
     </property>
		<property dictRef="me:epsilon">
      <scalar>506</scalar>
     </property>
     <property dictRef="me:sigma">
      <scalar>2.71</scalar>
     </property>
     <property dictRef="me:spinMultiplicity">
      <scalar>2</scalar>
     </property>
   </propertyList>

<me:DOSCMethod>QMRotors</me:DOSCMethod>

		<me:energyTransferModel xsi:type="me:ExponentialDown">
		<me:deltaEDown bathGas="N2" units="cm-1" lower="125" upper="2600" stepsize="10">1597</me:deltaEDown>
		<me:deltaEDownTExponent bathGas="N2" referenceTemperature="298" >0.25</me:deltaEDownTExponent>

		<me:deltaEDown bathGas="O2" units="cm-1" lower="125" upper="2600" stepsize="10">977</me:deltaEDown>
		<me:deltaEDownTExponent bathGas="O2" referenceTemperature="298" >0.25</me:deltaEDownTExponent>

   </me:energyTransferModel>

  </molecule>


		<molecule id="N2">
   <atom elementType="N"/>
   <propertyList>
     <property dictRef="me:epsilon">
      <scalar>48.0</scalar>
     </property>
     <property dictRef="me:sigma">
      <scalar>3.90</scalar>
     </property>
     <property dictRef="me:MW">
      <scalar units="amu">28.0</scalar>
     </property>
   </propertyList>
  </molecule>
```

```
  <molecule id="He">
    <atom elementType="He"/>
    <propertyList>
      <property dictRef="me:epsilon">
        <scalar>10.22</scalar>
      </property>
      <property dictRef="me:sigma">
        <scalar>2.511</scalar>
      </property>
      <property dictRef="me:MW">
        <scalar>4.04</scalar>
      </property>
    </propertyList>
  </molecule>

                <molecule id="Ar">
    <atom elementType="Ar"/>
    <propertyList>
      <property dictRef="me:epsilon">
        <scalar>114</scalar>
      </property>
      <property dictRef="me:sigma">
        <scalar>3.47</scalar>
      </property>
      <property dictRef="me:MW">
        <scalar>39.948</scalar>
      </property>
    </propertyList>
  </molecule>

  <molecule id="O2">
    <atom elementType="O"/>
    <propertyList>
      <property dictRef="me:epsilon">
        <scalar>74.5</scalar>
      </property>
      <property dictRef="me:sigma">
        <scalar>3.80</scalar>
      </property>
      <property dictRef="me:MW">
        <scalar units="amu">32.0</scalar>
      </property>
    </propertyList>
  </molecule>


</moleculeList>
<reactionList>

  <reaction id="R1">
    <reactant>
      <molecule ref="HO2" role="deficientReactant" />
    </reactant>
    <reactant>
      <molecule ref="NO2" role="excessReactant" />
    </reactant>
    <product>
      <molecule ref="HOONO2" role="modelled" />
    </product>
    <me:MCRCMethod xsi:type="me:MesmerILT">

      <me:preExponential lower="1e-13" upper="5e-11" stepsize="1e-13">5.18e-12</me:preExponential>
      <me:activationEnergy units="kJ/mol">0.0</me:activationEnergy>
      <me:TInfinity>298.0</me:TInfinity>
   <me:nInfinity lower="-1.9" upper="1.9" stepsize="0.005">-1.249</me:nInfinity>
    </me:MCRCMethod>
    <me:excessReactantConc>1e15</me:excessReactantConc>
  </reaction>

</reactionList>
<me:conditions>
```

```
<me:bathGas>N2</me:bathGas>
<me:PTs>

<!--Kurylo 1968 90 441 -->

<me:PTpair units="Torr" P= " 25 " T= "298" precision="d"> <me:bathGas>N2</me:bathGas> <me:experimentalRate ref1= "HO2" ref2= "HO2" refReaction="R1" error=" 1.21E-14 "> 1.21E-13 </me:experimentalRate> </me:PTpair>
<me:PTpair units="Torr" P= "50 " T= "298" precision="d"> <me:bathGas>N2</me:bathGas> <me:experimentalRate ref1= "HO2" ref2= "HO2" refReaction="R1" error=" 2.32E-14 "> 2.32E-13 </me:experimentalRate> </me:PTpair>
<me:PTpair units="Torr" P= "100" T= "298" precision="d"> <me:bathGas>N2</me:bathGas> <me:experimentalRate ref1= "HO2" ref2="HO2"refReaction="R1" error="3.49E-14">3.49E-13</me:experimentalRate></me:PTpair>
<me:PTpair units="Torr" P= "200" T= "298" precision="d"> <me:bathGas>N2</me:bathGas> <me:experimentalRate ref1= "HO2" ref2= "HO2" refReaction="R1" error=" 6.25E-14 ">6.25E-13 </me:experimentalRate> </me:PTpair>
<me:PTpair units="Torr" P= "400" T= "298" precision="d"> <me:bathGas>N2</me:bathGas> <me:experimentalRate ref1= "HO2" ref2= "HO2" refReaction="R1" error=" 9.19E-14 "> 9.19E-13 </me:experimentalRate> </me:PTpair>
<me:PTpair units="Torr" P= "600" T= " 298 " precision="d"> <me:bathGas>N2</me:bathGas> <me:experimentalRate ref1= "HO2" ref2="HO2" refReaction="R1" error="1.179E-13">1.179E-12</me:experimentalRate></me:PTpair>
<me:PTpair units="Torr" P= "25" T= "298" precision="d"> <me:bathGas>O2</me:bathGas> <me:experimentalRate ref1= "HO2" ref2= "HO2" refReaction="R1" error=" 1.03E-14 "> 1.03E-13 </me:experimentalRate></me:PTpair>
<me:PTpair units="Torr" P= "50" T= "298 " precision="d"> <me:bathGas>O2</me:bathGas> <me:experimentalRate ref1="HO2" ref2= "HO2" refReaction="R1" error="1.76E-14">1.76E-13 </me:experimentalRate></me:PTpair>
<me:PTpair units="Torr" P= "100" T= "298" precision="d"> <me:bathGas>O2</me:bathGas> <me:experimentalRate ref1="HO2" ref2="HO2"refReaction="R1" error="2.98E-14">2.98E-13</me:experimentalRate></me:PTpair>
<me:PTpair units="Torr" P= "200" T= "298" precision="d"> <me:bathGas>O2</me:bathGas> <me:experimentalRate ref1="HO2" ref2= "HO2"refReaction="R1" error=" 5.21E-14 "> 5.21E-13 </me:experimentalRate> </me:PTpair>
<me:PTpair units="Torr" P= "400" T= "298" precision="d"> <me:bathGas>O2</me:bathGas><me:experimentalRate ref1="HO2" ref2="HO2"refReaction="R1" error="8.1E-14">8.1E-13</me:experimentalRate></me:PTpair>
<me:PTpair units="Torr" P= "600" T= "298" precision="d"><me:bathGas>O2</me:bathGas><me:experimentalRate ref1="HO2"ref2="HO2" refReaction="R1" error="1.12E-13">1.12E-12</me:experimentalRate></me:PTpair>

<!--Kurylo 1968 90 441 1987 -->

<me:PTpair units="Torr" P= " 25" T= "358" precision="d"><me:bathGas>N2</me:bathGas><me:experimentalRate ref1="HO2" ref2="HO2" refReaction="R1" error="9E-15"> 6E-14 </me:experimentalRate> </me:PTpair>
<me:PTpair units="Torr" P= " 50" T= "358" precision="d"><me:bathGas>N2</me:bathGas><me:experimentalRate ref1="HO2" ref2="HO2" refReaction="R1" error="1.52E-14">1.02E-13</me:experimentalRate></me:PTpair>
<me:PTpair units="Torr" P= " 100" T= "358" precision="d"><me:bathGas>N2</me:bathGas><me:experimentalRate ref1="HO2" ref2="HO2" refReaction="R1" error="2.5E-14">1.92E-13</me:experimentalRate></me:PTpair>
<me:PTpair units="Torr" P= " 25" T= "337" precision="d"><me:bathGas>N2</me:bathGas><me:experimentalRate ref1="HO2" ref2="HO2" refReaction="R1" error="8.1E-15">8.1E-14</me:experimentalRate></me:PTpair>
<me:PTpair units="Torr" P= " 50" T= "337" precision="d"><me:bathGas>N2</me:bathGas><me:experimentalRate ref1="HO2" ref2="HO2" refReaction="R1" error="1.46E-14">1.46E-13</me:experimentalRate></me:PTpair>
<me:PTpair units="Torr" P= " 100" T= "337" precision="d"><me:bathGas>N2</me:bathGas><me:experimentalRate ref1="HO2" ref2="HO2" refReaction="R1" error="3.1E-14">2.37E-13</me:experimentalRate></me:PTpair>
<me:PTpair units="Torr" P= " 25" T= "317" precision="d"><me:bathGas>N2</me:bathGas><me:experimentalRate ref1="HO2" ref2="HO2" refReaction="R1" error="1.03E-14">1.03E-13</me:experimentalRate></me:PTpair>
<me:PTpair units="Torr" P= " 50" T= "317" precision="d"><me:bathGas>N2</me:bathGas><me:experimentalRate ref1="HO2" ref2="HO2" refReaction="R1" error="1.97E-14">1.97E-13</me:experimentalRate></me:PTpair>
<me:PTpair units="Torr" P= " 100" T= "317" precision="d"><me:bathGas>N2</me:bathGas><me:experimentalRate ref1="HO2" ref2="HO2" refReaction="R1" error="3.19E-14">3.19E-13</me:experimentalRate></me:PTpair>

<me:PTpair units="Torr" P= " 25" T= "298" precision="d"><me:bathGas>N2</me:bathGas><me:experimentalRate ref1="HO2" ref2="HO2" refReaction="R1" error="1.41E-14">1.21E-13</me:experimentalRate></me:PTpair>
<me:PTpair units="Torr" P= " 50" T= "298" precision="d"><me:bathGas>N2</me:bathGas><me:experimentalRate ref1="HO2" ref2="HO2" refReaction="R1" error="5.6E-14">2.32E-13</me:experimentalRate></me:PTpair>
<me:PTpair units="Torr" P= " 100" T= "298" precision="d"><me:bathGas>N2</me:bathGas><me:experimentalRate ref1="HO2" ref2="HO2" refReaction="R1" error="9.3E-14">3.49E-13</me:experimentalRate></me:PTpair>

<me:PTpair units="Torr" P= " 25" T= "283" precision="d"><me:bathGas>N2</me:bathGas><me:experimentalRate ref1="HO2" ref2="HO2" refReaction="R1" error="1.62E-14">1.62E-13</me:experimentalRate></me:PTpair>
<me:PTpair units="Torr" P= " 50" T= "283" precision="d"><me:bathGas>N2</me:bathGas><me:experimentalRate ref1="HO2" ref2="HO2" refReaction="R1" error="2.8E-14">2.8E-13</me:experimentalRate></me:PTpair>
<me:PTpair units="Torr" P= " 100" T= "283" precision="d"><me:bathGas>N2</me:bathGas><me:experimentalRate ref1="HO2" ref2="HO2" refReaction="R1" error="5.0E-14">4.89E-13</me:experimentalRate></me:PTpair>
<me:PTpair units="Torr" P= " 25" T= "263" precision="d"><me:bathGas>N2</me:bathGas><me:experimentalRate ref1="HO2" ref2="HO2" refReaction="R1" error="4.2E-14">2.39E-13</me:experimentalRate></me:PTpair>
<me:PTpair units="Torr" P= " 50" T= "263" precision="d"><me:bathGas>N2</me:bathGas><me:experimentalRate ref1="HO2" ref2="HO2" refReaction="R1" error="5.5E-14">3.77E-13</me:experimentalRate></me:PTpair>
<me:PTpair units="Torr" P= " 100" T= "263" precision="d"><me:bathGas>N2</me:bathGas><me:experimentalRate ref1="HO2" ref2="HO2" refReaction="R1" error="8.4E-14">6.69E-13</me:experimentalRate></me:PTpair>
<me:PTpair units="Torr" P= " 25" T= "244" precision="d"><me:bathGas>N2</me:bathGas><me:experimentalRate ref1="HO2" ref2="HO2" refReaction="R1" error="6.04E-14">3.02E-13</me:experimentalRate></me:PTpair>
<me:PTpair units="Torr" P= " 50" T= "244" precision="d"><me:bathGas>N2</me:bathGas><me:experimentalRate ref1="HO2" ref2="HO2" refReaction="R1" error="5.4E-14">4.55E-13</me:experimentalRate></me:PTpair>
<me:PTpair units="Torr" P= " 100" T= "244" precision="d"><me:bathGas>N2</me:bathGas><me:experimentalRate ref1="HO2" ref2="HO2" refReaction="R1" error="1.32E-13">7.2E-13</me:experimentalRate></me:PTpair>
<me:PTpair units="Torr" P= " 25" T= "228" precision="d"><me:bathGas>N2</me:bathGas><me:experimentalRate ref1="HO2" ref2="HO2" refReaction="R1" error="9.3E-14">3.65E-13</me:experimentalRate></me:PTpair>
<me:PTpair units="Torr" P= " 50" T= "228" precision="d"><me:bathGas>N2</me:bathGas><me:experimentalRate ref1="HO2" ref2="HO2" refReaction="R1" error="1.64E-13">5.76E-13</me:experimentalRate></me:PTpair>
<me:PTpair units="Torr" P= " 100" T= "228" precision="d"><me:bathGas>N2</me:bathGas><me:experimentalRate ref1="HO2" ref2="HO2" refReaction="R1" error="1.98E-13">8.46E-13</me:experimentalRate></me:PTpair>

<!--Sander 1984 -->

<me:PTpair units="PPCC" P= "3.23E18" T= "298" precision="d"><me:bathGas>N2</me:bathGas><me:experimentalRate ref1="HO2" ref2="HO2"refReaction="R1" error="0.46E-13">4.62E-13</me:experimentalRate></me:PTpair>
<me:PTpair units="PPCC" P= "6.46E18" T= "298" precision="d"><me:bathGas>N2</me:bathGas><me:experimentalRate ref1="HO2" ref2="HO2"refReaction="R1" error="0.65E-13">6.45E-13</me:experimentalRate></me:PTpair>
<me:PTpair units="PPCC" P= "1.13E19" T= "298" precision="d"><me:bathGas>N2</me:bathGas><me:experimentalRate ref1="HO2" ref2="HO2"refReaction="R1" error="0.94E-13">9.44E-13</me:experimentalRate></me:PTpair>
<me:PTpair units="PPCC" P= "1.62E19" T= "298" precision="d"><me:bathGas>N2</me:bathGas><me:experimentalRate ref1="HO2" ref2="HO2"refReaction="R1" error="0.11E-12">1.12E-12</me:experimentalRate></me:PTpair>
<me:PTpair units="PPCC" P= "2.26E19" T= "298" precision="d"><me:bathGas>N2</me:bathGas><me:experimentalRate ref1="HO2" ref2="HO2"refReaction="R1" error="0.13E-12">1.32E-12</me:experimentalRate></me:PTpair>
<me:PTpair units="PPCC" P= "1.68E18" T= "298" precision="d"><me:bathGas>N2</me:bathGas><me:experimentalRate ref1="HO2" ref2="HO2"refReaction="R1" error="0.53E-13">3.05E-13</me:experimentalRate></me:PTpair>

<!--Bacak and Percival -->

<!-- <me:PTpair units="Torr" P= " 200" T= "298" precision="d"><me:bathGas>N2</me:bathGas><me:experimentalRate ref1="HO2" ref2="HO2" refReaction="R1" error="0.63E-13">6.32E-13</me:experimentalRate></me:PTpair> -->
<!-- <me:PTpair units="Torr" P= " 300" T= "298" precision="d"><me:bathGas>N2</me:bathGas><me:experimentalRate ref1="HO2" ref2="HO2" refReaction="R1" error="0.80E-13">8.02E-13</me:experimentalRate></me:PTpair> -->
<!-- <me:PTpair units="Torr" P= " 418" T= "298" precision="d"><me:bathGas>N2</me:bathGas><me:experimentalRate ref1="HO2" ref2="HO2" refReaction="R1" error="0.97E-13">9.67E-13</me:experimentalRate></me:PTpair> -->
<!-- <me:PTpair units="Torr" P= " 517" T= "298" precision="d"><me:bathGas>N2</me:bathGas><me:experimentalRate ref1="HO2" ref2="HO2" refReaction="R1" error="0.11E-12">1.10E-12</me:experimentalRate></me:PTpair> -->
<!-- <me:PTpair units="Torr" P= " 607" T= "298" precision="d"><me:bathGas>N2</me:bathGas><me:experimentalRate ref1="HO2" ref2="HO2" refReaction="R1" error="0.12E-12">1.21E-12</me:experimentalRate></me:PTpair> -->
<!-- <me:PTpair units="Torr" P= " 712" T= "298" precision="d"><me:bathGas>N2</me:bathGas><me:experimentalRate ref1="HO2" ref2="HO2" refReaction="R1" error="0.13E-12">1.31E-12</me:experimentalRate></me:PTpair> -->

<!-- <me:PTpair units="Torr" P= " 300" T= "223" precision="d"><me:bathGas>N2</me:bathGas><me:experimentalRate ref1="HO2" ref2="HO2" refReaction="R1" error="0.10E-12">1.03E-12</me:experimentalRate></me:PTpair> -->
<!-- <me:PTpair units="Torr" P= " 400" T= "223" precision="d"><me:bathGas>N2</me:bathGas><me:experimentalRate ref1="HO2" ref2="HO2" refReaction="R1" error="0.12E-12">1.15E-12</me:experimentalRate></me:PTpair> -->
<!-- <me:PTpair units="Torr" P= " 500" T= "223" precision="d"><me:bathGas>N2</me:bathGas><me:experimentalRate ref1="HO2" ref2="HO2" refReaction="R1" error="0.14E-12">1.32E-12</me:experimentalRate></me:PTpair> -->
<!-- <me:PTpair units="Torr" P= " 600" T= "223" precision="d"><me:bathGas>N2</me:bathGas><me:experimentalRate ref1="HO2" ref2="HO2" refReaction="R1" error="0.14E-12">1.43E-12</me:experimentalRate></me:PTpair> -->

<!-- <me:PTpair units="Torr" P= " 150" T= "200" precision="d"><me:bathGas>N2</me:bathGas><me:experimentalRate ref1="HO2" ref2="HO2" refReaction="R1" error="0.73E-13">7.27E-13</me:experimentalRate></me:PTpair> -->
<!-- <me:PTpair units="Torr" P= " 300" T= "200" precision="d"><me:bathGas>N2</me:bathGas><me:experimentalRate ref1="HO2" ref2="HO2" refReaction="R1" error="0.12E-12">1.17E-12</me:experimentalRate></me:PTpair> -->
<!-- <me:PTpair units="Torr" P= " 400" T= "200" precision="d"><me:bathGas>N2</me:bathGas><me:experimentalRate ref1="HO2" ref2="HO2" refReaction="R1" error="0.15E-12">1.45E-12</me:experimentalRate></me:PTpair> -->
<!-- <me:PTpair units="Torr" P= " 600" T= "200" precision="d"><me:bathGas>N2</me:bathGas><me:experimentalRate ref1="HO2" ref2="HO2" refReaction="R1" error="0.16E-12">1.65E-12</me:experimentalRate></me:PTpair> -->
<!-- <me:PTpair units="Torr" P= " 700" T= "200" precision="d"><me:bathGas>N2</me:bathGas><me:experimentalRate ref1="HO2" ref2="HO2" refReaction="R1" error="0.18E-12">1.78E-12</me:experimentalRate> </me:PTpair> -->

<!--Christenson - Sander et al errors times 1.83 -->

<me:PTpair units="PPCC" P= " 3.24E18" T= "298" precision="d"><me:bathGas>N2 </me:bathGas><me:experimentalRate ref1="HO2"ref2="HO2" refReaction="R1" error="1.83E-14">4E13</me:experimentalRate></me:PTpair>
<me:PTpair units="PPCC" P= " 1.45E18" T= "295" precision="d"><me:bathGas>N2 </me:bathGas><me:experimentalRate ref1="HO2"ref2="HO2" refReaction="R1" error="1.83E-14">2.2E-13</me:experimentalRate></me:PTpair>
<me:PTpair units="PPCC" P= " 1.69E18" T= "295" precision="d"><me:bathGas>N2 </me:bathGas><me:experimentalRate ref1="HO2"ref2="HO2" refReaction="R1" error="3.66E-14">2.5E-13</me:experimentalRate></me:PTpair>
<me:PTpair units="PPCC" P= " 1.97E18" T= "295" precision="d"><me:bathGas>N2 </me:bathGas><me:experimentalRate ref1="HO2"ref2="HO2" refReaction="R1" error="1.83E-14">2.8E-13</me:experimentalRate></me:PTpair>
<me:PTpair units="PPCC" P= " 1.97E18" T= "295" precision="d"><me:bathGas>N2 </me:bathGas><me:experimentalRate ref1="HO2"ref2="HO2" refReaction="R1" error="1.83E-14">2.8E-13</me:experimentalRate></me:PTpair>
<me:PTpair units="PPCC" P= " 2.32E18" T= "295" precision="d"><me:bathGas>N2 </me:bathGas><me:experimentalRate ref1="HO2"ref2="HO2" refReaction="R1" error="1.83E-14">3.2E-13</me:experimentalRate></me:PTpair>
<me:PTpair units="PPCC" P= " 2.66E18" T= "295" precision="d"><me:bathGas>N2 </me:bathGas><me:experimentalRate ref1="HO2"ref2="HO2" refReaction="R1" error="1.83E-14">3.4E-13</me:experimentalRate></me:PTpair>
<me:PTpair units="PPCC" P= " 2.96E18" T= "295" precision="d"><me:bathGas>N2 </me:bathGas><me:experimentalRate ref1="HO2"ref2="HO2" refReaction="R1" error="3.66E-14">3.9E-13</me:experimentalRate></me:PTpair>
<me:PTpair units="PPCC" P= " 3.21E18" T= "295" precision="d"><me:bathGas>N2 </me:bathGas><me:experimentalRate ref1="HO2"ref2="HO2" refReaction="R1" error="5.49E-14">4.1E-13</me:experimentalRate></me:PTpair>
<me:PTpair units="PPCC" P= " 3.54E18" T= "295" precision="d"><me:bathGas>N2 </me:bathGas><me:experimentalRate ref1="HO2"ref2="HO2" refReaction="R1" error="3.66E-14">4.3E-13</me:experimentalRate></me:PTpair>
<me:PTpair units="PPCC" P= " 3.56E18" T= "295" precision="d"><me:bathGas>N2 </me:bathGas><me:experimentalRate ref1="HO2"ref2="HO2" refReaction="R1" error="3.66E-14">4.2E-13</me:experimentalRate></me:PTpair>
<me:PTpair units="PPCC" P= " 3.6E18" T= "295" precision="d"><me:bathGas>N2 </me:bathGas><me:experimentalRate ref1="HO2"ref2="HO2" refReaction="R1" error="1.83E-14">4.2E-13</me:experimentalRate></me:PTpair>
<me:PTpair units="PPCC" P= " 3.62E18" T= "295" precision="d"><me:bathGas>N2 </me:bathGas><me:experimentalRate ref1="HO2"ref2="HO2" refReaction="R1" error="1.83E-14">4.4E-13</me:experimentalRate></me:PTpair>
<me:PTpair units="PPCC" P= " 4.11E18" T= "295" precision="d"><me:bathGas>N2 </me:bathGas><me:experimentalRate ref1="HO2"ref2="HO2" refReaction="R1" error="1.83E-14">4.5E-13</me:experimentalRate></me:PTpair>
<me:PTpair units="PPCC" P= " 4.66E18" T= "295" precision="d"><me:bathGas>N2 </me:bathGas><me:experimentalRate ref1="HO2"ref2="HO2" refReaction="R1" error="7.32E-14">5E-13</me:experimentalRate></me:PTpair>
<me:PTpair units="PPCC" P= " 5.01E18" T= "295" precision="d"><me:bathGas>N2 </me:bathGas><me:experimentalRate ref1="HO2"ref2="HO2" refReaction="R1" error="5.49E-14">5.7E-13</me:experimentalRate></me:PTpair>
<me:PTpair units="PPCC" P= " 5.04E18" T= "295" precision="d"><me:bathGas>N2 </me:bathGas><me:experimentalRate ref1="HO2"ref2="HO2" refReaction="R1" error="3.66E-14">5.2E-13</me:experimentalRate></me:PTpair>
<me:PTpair units="PPCC" P= " 5.39E18" T= "295" precision="d"><me:bathGas>N2 </me:bathGas><me:experimentalRate ref1="HO2"ref2="HO2" refReaction="R1" error="5.49E-14">6.1E-13</me:experimentalRate></me:PTpair>
<me:PTpair units="PPCC" P= " 6.36E18" T= "295" precision="d"><me:bathGas>N2 </me:bathGas><me:experimentalRate ref1="HO2"ref2="HO2" refReaction="R1" error="1.464E-13">5.8E-13</me:experimentalRate></me:PTpair>

<me:PTpair units="PPCC" P= " 3.35E18" T= "288" precision="d"><me:bathGas>N2 </me:bathGas><me:experimentalRate ref1="HO2"ref2="HO2" refReaction="R1" error="1.83E-14">4.4E-13</me:experimentalRate></me:PTpair>
<me:PTpair units="PPCC" P= " 1.24E18" T= "284" precision="d"><me:bathGas>N2 </me:bathGas><me:experimentalRate ref1="HO2"ref2="HO2" refReaction="R1" error="1.83E-14">2.1E-13</me:experimentalRate></me:PTpair>
<me:PTpair units="PPCC" P= " 1.75E18" T= "284" precision="d"><me:bathGas>N2 </me:bathGas><me:experimentalRate ref1="HO2"ref2="HO2" refReaction="R1" error="1.83E-14">2.8E-13</me:experimentalRate></me:PTpair>
<me:PTpair units="PPCC" P= " 2.18E18" T= "284" precision="d"><me:bathGas>N2 </me:bathGas><me:experimentalRate ref1="HO2"ref2="HO2" refReaction="R1" error="1.83E-14">3.1E-13</me:experimentalRate></me:PTpair>
<me:PTpair units="PPCC" P= " 2.65E18" T= "284" precision="d"><me:bathGas>N2 </me:bathGas><me:experimentalRate ref1="HO2"ref2="HO2" refReaction="R1" error="1.83E-14">3.6E-13</me:experimentalRate></me:PTpair>
<me:PTpair units="PPCC" P= " 2.89E18" T= "284" precision="d"><me:bathGas>N2 </me:bathGas><me:experimentalRate ref1="HO2"ref2="HO2" refReaction="R1" error="1.83E-14">3.8E-13</me:experimentalRate></me:PTpair>
<me:PTpair units="PPCC" P= " 3.22E18" T= "284" precision="d"><me:bathGas>N2 </me:bathGas><me:experimentalRate ref1="HO2"ref2="HO2" refReaction="R1" error="1.83E-14">4.1E-13</me:experimentalRate></me:PTpair>
<me:PTpair units="PPCC" P= " 3.59E18" T= "284" precision="d"><me:bathGas>N2 </me:bathGas><me:experimentalRate ref1="HO2"ref2="HO2" refReaction="R1" error="1.83E-14">4.4E-13</me:experimentalRate></me:PTpair>
<me:PTpair units="PPCC" P= " 4.09E18" T= "284" precision="d"><me:bathGas>N2 </me:bathGas><me:experimentalRate ref1="HO2"ref2="HO2" refReaction="R1" error="1.83E-14">4.5E-13</me:experimentalRate></me:PTpair>
<me:PTpair units="PPCC" P= " 4.57E18" T= "284" precision="d"><me:bathGas>N2 </me:bathGas><me:experimentalRate ref1="HO2"ref2="HO2" refReaction="R1" error="3.66E-14">5E-13</me:experimentalRate></me:PTpair>
<me:PTpair units="PPCC" P= " 4.84E18" T= "284" precision="d"><me:bathGas>N2 </me:bathGas><me:experimentalRate ref1="HO2"ref2="HO2" refReaction="R1" error="1.83E-14">5E-13</me:experimentalRate></me:PTpair>
<me:PTpair units="PPCC" P= " 5.21E18" T= "284" precision="d"><me:bathGas>N2 </me:bathGas><me:experimentalRate ref1="HO2"ref2="HO2" refReaction="R1" error="3.66E-14">5.4E-13</me:experimentalRate></me:PTpair>
<me:PTpair units="PPCC" P= " 3.53E18" T= "273" precision="d"><me:bathGas>N2 </me:bathGas><me:experimentalRate ref1="HO2"ref2="HO2" refReaction="R1" error="1.83E-14">5.1E-13</me:experimentalRate></me:PTpair>
<me:PTpair units="PPCC" P= " 1.48E18" T= "272" precision="d"><me:bathGas>N2 </me:bathGas><me:experimentalRate ref1="HO2"ref2="HO2" refReaction="R1" error="1.83E-14">2.6E-13</me:experimentalRate></me:PTpair>
<me:PTpair units="PPCC" P= " 1.98E18" T= "272" precision="d"><me:bathGas>N2 </me:bathGas><me:experimentalRate ref1="HO2"ref2="HO2" refReaction="R1" error="1.83E-14">3.3E-13</me:experimentalRate></me:PTpair>
```

```
<me:PTpair units="PPCC" P= " 2.36E18" T= "272" precision="d"><me:bathGas>N2 </me:bathGas><me:experimentalRate ref1="HO2"ref2="HO2" refReaction="R1" error="1.83E-14">3.9E-13</me:experimentalRate></me:PTpair>
<me:PTpair units="PPCC" P= " 2.85E18" T= "272" precision="d"><me:bathGas>N2 </me:bathGas><me:experimentalRate ref1="HO2"ref2="HO2" refReaction="R1" error="1.83E-14">4.3E-13</me:experimentalRate></me:PTpair>
<me:PTpair units="PPCC" P= " 3.21E18" T= "272" precision="d"><me:bathGas>N2 </me:bathGas><me:experimentalRate ref1="HO2"ref2="HO2" refReaction="R1" error="1.83E-14">4.6E-13</me:experimentalRate></me:PTpair>
<me:PTpair units="PPCC" P= " 3.55E18" T= "272" precision="d"><me:bathGas>N2 </me:bathGas><me:experimentalRate ref1="HO2"ref2="HO2" refReaction="R1" error="1.83E-14">5.1E-13</me:experimentalRate></me:PTpair>
<me:PTpair units="PPCC" P= " 3.9E18" T= "272" precision="d"><me:bathGas>N2 </me:bathGas><me:experimentalRate ref1="HO2"ref2="HO2" refReaction="R1" error="1.83E-14">5.4E-13</me:experimentalRate></me:PTpair>
<me:PTpair units="PPCC" P= " 4.31E18" T= "272" precision="d"><me:bathGas>N2 </me:bathGas><me:experimentalRate ref1="HO2"ref2="HO2" refReaction="R1" error="3.66E-14">5.5E-13</me:experimentalRate></me:PTpair>
<me:PTpair units="PPCC" P= " 5.09E18" T= "272" precision="d"><me:bathGas>N2 </me:bathGas><me:experimentalRate ref1="HO2"ref2="HO2" refReaction="R1" error="1.83E-14">5.8E-13</me:experimentalRate></me:PTpair>
<me:PTpair units="PPCC" P= " 5.46E18" T= "272" precision="d"><me:bathGas>N2 </me:bathGas><me:experimentalRate ref1="HO2"ref2="HO2" refReaction="R1" error="5.49E-14">6.5E-13</me:experimentalRate></me:PTpair>

<me:PTpair units="PPCC" P= " 1.33E18" T= "258" precision="d"><me:bathGas>N2 </me:bathGas><me:experimentalRate ref1="HO2"ref2="HO2" refReaction="R1" error="1.83E-14">2.9E-13</me:experimentalRate></me:PTpair>
<me:PTpair units="PPCC" P= " 1.67E18" T= "258" precision="d"><me:bathGas>N2 </me:bathGas><me:experimentalRate ref1="HO2"ref2="HO2" refReaction="R1" error="1.83E-14">3.6E-13</me:experimentalRate></me:PTpair>
<me:PTpair units="PPCC" P= " 2.26E18" T= "258" precision="d"><me:bathGas>N2 </me:bathGas><me:experimentalRate ref1="HO2"ref2="HO2" refReaction="R1" error="1.83E-14">4.3E-13</me:experimentalRate></me:PTpair>
<me:PTpair units="PPCC" P= " 2.41E18" T= "258" precision="d"><me:bathGas>N2 </me:bathGas><me:experimentalRate ref1="HO2"ref2="HO2" refReaction="R1" error="1.83E-14">4.5E-13</me:experimentalRate></me:PTpair>
<me:PTpair units="PPCC" P= " 2.99E18" T= "258" precision="d"><me:bathGas>N2 </me:bathGas><me:experimentalRate ref1="HO2"ref2="HO2" refReaction="R1" error="1.83E-14">5.6E-13</me:experimentalRate></me:PTpair>
<me:PTpair units="PPCC" P= " 3.06E18" T= "258" precision="d"><me:bathGas>N2 </me:bathGas><me:experimentalRate ref1="HO2"ref2="HO2" refReaction="R1" error="1.83E-14">5.4E-13</me:experimentalRate></me:PTpair>
<me:PTpair units="PPCC" P= " 3.38E18" T= "258" precision="d"><me:bathGas>N2 </me:bathGas><me:experimentalRate ref1="HO2"ref2="HO2" refReaction="R1" error="1.83E-14">5.7E-13</me:experimentalRate></me:PTpair>
<me:PTpair units="PPCC" P= " 3.66E18" T= "258" precision="d"><me:bathGas>N2 </me:bathGas><me:experimentalRate ref1="HO2"ref2="HO2" refReaction="R1" error="7.32E-14">6E-13</me:experimentalRate></me:PTpair>
<me:PTpair units="PPCC" P= " 3.84E18" T= "258" precision="d"><me:bathGas>N2 </me:bathGas><me:experimentalRate ref1="HO2"ref2="HO2" refReaction="R1" error="1.83E-14">6.4E-13</me:experimentalRate></me:PTpair>
<me:PTpair units="PPCC" P= " 4.23E18" T= "258" precision="d"><me:bathGas>N2 </me:bathGas><me:experimentalRate ref1="HO2"ref2="HO2" refReaction="R1" error="1.098E-13">6.4E-13</me:experimentalRate></me:PTpair>
<me:PTpair units="PPCC" P= " 3.86E18" T= "250" precision="d"><me:bathGas>N2 </me:bathGas><me:experimentalRate ref1="HO2"ref2="HO2" refReaction="R1" error="1.83E-14">6.7E-13</me:experimentalRate></me:PTpair>
<me:PTpair units="PPCC" P= " 1.68E18" T= "243" precision="d"><me:bathGas>N2 </me:bathGas><me:experimentalRate ref1="HO2"ref2="HO2" refReaction="R1" error="5.49E-14">4.3E-13</me:experimentalRate></me:PTpair>
<me:PTpair units="PPCC" P= " 2.12E18" T= "243" precision="d"><me:bathGas>N2 </me:bathGas><me:experimentalRate ref1="HO2"ref2="HO2" refReaction="R1" error="3.66E-14">4.9E-13</me:experimentalRate></me:PTpair>
<me:PTpair units="PPCC" P= " 2.97E18" T= "243" precision="d"><me:bathGas>N2 </me:bathGas><me:experimentalRate ref1="HO2"ref2="HO2" refReaction="R1" error="5.49E-14">5.9E-13</me:experimentalRate></me:PTpair>
<me:PTpair units="PPCC" P= " 3.38E18" T= "243" precision="d"><me:bathGas>N2 </me:bathGas><me:experimentalRate ref1="HO2"ref2="HO2" refReaction="R1" error="3.66E-14">6.4E-13</me:experimentalRate></me:PTpair>
<me:PTpair units="PPCC" P= " 3.77E18" T= "243" precision="d"><me:bathGas>N2 </me:bathGas><me:experimentalRate ref1="HO2"ref2="HO2" refReaction="R1" error="3.66E-14">7.6E-13</me:experimentalRate></me:PTpair>
<me:PTpair units="PPCC" P= " 4.05E18" T= "243" precision="d"><me:bathGas>N2 </me:bathGas><me:experimentalRate ref1="HO2"ref2="HO2" refReaction="R1" error="3.66E-14">7.7E-13</me:experimentalRate></me:PTpair>
<me:PTpair units="PPCC" P= " 4.54E18" T= "243" precision="d"><me:bathGas>N2 </me:bathGas><me:experimentalRate ref1="HO2"ref2="HO2" refReaction="R1" error="5.49E-14">8.1E-13</me:experimentalRate></me:PTpair>
<me:PTpair units="PPCC" P= " 4.84E18" T= "243" precision="d"><me:bathGas>N2 </me:bathGas><me:experimentalRate ref1="HO2"ref2="HO2" refReaction="R1" error="3.66E-14">8.5E-13</me:experimentalRate></me:PTpair>
<me:PTpair units="PPCC" P= " 5.41E18" T= "243" precision="d"><me:bathGas>N2 </me:bathGas><me:experimentalRate ref1="HO2"ref2="HO2" refReaction="R1" error="9.15E-14">9.1E-13</me:experimentalRate></me:PTpair>
<me:PTpair units="PPCC" P= " 6E18" T= "243" precision="d"><me:bathGas>N2 </me:bathGas><me:experimentalRate ref1="HO2"ref2="HO2" refReaction="R1" error="5.49E-14">9.6E-13</me:experimentalRate></me:PTpair>
<me:PTpair units="PPCC" P= " 4.02E18" T= "240" precision="d"><me:bathGas>N2 </me:bathGas><me:experimentalRate ref1="HO2"ref2="HO2" refReaction="R1" error="1.83E-14">6.9E-13</me:experimentalRate></me:PTpair>

<me:PTpair units="PPCC" P= " 4.17E18" T= "231" precision="d"><me:bathGas>N2 </me:bathGas><me:experimentalRate ref1="HO2"ref2="HO2" refReaction="R1" error="5.49E-14">8.5E-13</me:experimentalRate></me:PTpair>
<me:PTpair units="PPCC" P= " 8.35E18" T= "231" precision="d"><me:bathGas>N2 </me:bathGas><me:experimentalRate ref1="HO2"ref2="HO2" refReaction="R1" error="7.32E-14">9.4E-13</me:experimentalRate></me:PTpair>
<me:PTpair units="PPCC" P= " 1.47E18" T= "231" precision="d"><me:bathGas>N2 </me:bathGas><me:experimentalRate ref1="HO2"ref2="HO2" refReaction="R1" error="1.098E-13">3.7E-13</me:experimentalRate></me:PTpair>
<me:PTpair units="PPCC" P= " 1.68E18" T= "231" precision="d"><me:bathGas>N2 </me:bathGas><me:experimentalRate ref1="HO2"ref2="HO2" refReaction="R1" error="1.098E-13">4.3E-13</me:experimentalRate></me:PTpair>
<me:PTpair units="PPCC" P= " 2.2E18" T= "231" precision="d"><me:bathGas>N2 </me:bathGas><me:experimentalRate ref1="HO2"ref2="HO2" refReaction="R1" error="1.098E-13">5.3E-13</me:experimentalRate></me:PTpair>
<me:PTpair units="PPCC" P= " 2.59E18" T= "231" precision="d"><me:bathGas>N2 </me:bathGas><me:experimentalRate ref1="HO2"ref2="HO2" refReaction="R1" error="1.464E-13">5.5E-13</me:experimentalRate></me:PTpair>
<me:PTpair units="PPCC" P= " 2.73E18" T= "231" precision="d"><me:bathGas>N2 </me:bathGas><me:experimentalRate ref1="HO2"ref2="HO2" refReaction="R1" error="1.098E-13">5.9E-13</me:experimentalRate></me:PTpair>
<me:PTpair units="PPCC" P= " 3.15E18" T= "231" precision="d"><me:bathGas>N2 </me:bathGas><me:experimentalRate ref1="HO2"ref2="HO2" refReaction="R1" error="1.098E-13">6.4E-13</me:experimentalRate></me:PTpair>
<me:PTpair units="PPCC" P= " 3.7E18" T= "231" precision="d"><me:bathGas>N2 </me:bathGas><me:experimentalRate ref1="HO2"ref2="HO2" refReaction="R1" error="1.281E-13">7.4E-13</me:experimentalRate></me:PTpair>
<me:PTpair units="PPCC" P= " 3.84E18" T= "231" precision="d"><me:bathGas>N2 </me:bathGas><me:experimentalRate ref1="HO2"ref2="HO2" refReaction="R1" error="1.464E-13">8.5E-13</me:experimentalRate></me:PTpair>
<me:PTpair units="PPCC" P= " 4.33E18" T= "231" precision="d"><me:bathGas>N2 </me:bathGas><me:experimentalRate ref1="HO2"ref2="HO2" refReaction="R1" error="1.464E-13">8.2E-13</me:experimentalRate></me:PTpair>
<me:PTpair units="PPCC" P= " 4.77E18" T= "231" precision="d"><me:bathGas>N2 </me:bathGas><me:experimentalRate ref1="HO2"ref2="HO2" refReaction="R1" error="1.281E-13">9.5E-13</me:experimentalRate></me:PTpair>
<me:PTpair units="PPCC" P= " 1.57E18" T= "219" precision="d"><me:bathGas>N2 </me:bathGas><me:experimentalRate ref1="HO2"ref2="HO2" refReaction="R1" error="1.464E-13">4.1E-13</me:experimentalRate></me:PTpair>
<me:PTpair units="PPCC" P= " 1.78E18" T= "219" precision="d"><me:bathGas>N2 </me:bathGas><me:experimentalRate ref1="HO2"ref2="HO2" refReaction="R1" error="1.647E-13">4.8E-13</me:experimentalRate></me:PTpair>
<me:PTpair units="PPCC" P= " 2.02E18" T= "219" precision="d"><me:bathGas>N2 </me:bathGas><me:experimentalRate ref1="HO2"ref2="HO2" refReaction="R1" error="1.464E-13">5.3E-13</me:experimentalRate></me:PTpair>
<me:PTpair units="PPCC" P= " 2.44E18" T= "219" precision="d"><me:bathGas>N2 </me:bathGas><me:experimentalRate ref1="HO2"ref2="HO2" refReaction="R1" error="1.464E-13">6.1E-13</me:experimentalRate></me:PTpair>
<me:PTpair units="PPCC" P= " 2.82E18" T= "219" precision="d"><me:bathGas>N2 </me:bathGas><me:experimentalRate ref1="HO2"ref2="HO2" refReaction="R1" error="1.647E-13">6.9E-13</me:experimentalRate></me:PTpair>
<me:PTpair units="PPCC" P= " 3.12E18" T= "219" precision="d"><me:bathGas>N2 </me:bathGas><me:experimentalRate ref1="HO2"ref2="HO2" refReaction="R1" error="1.647E-13">8.2E-13</me:experimentalRate></me:PTpair>
<me:PTpair units="PPCC" P= " 3.28E18" T= "219" precision="d"><me:bathGas>N2 </me:bathGas><me:experimentalRate ref1="HO2"ref2="HO2" refReaction="R1" error="1.647E-13">7.1E-13</me:experimentalRate></me:PTpair>
<me:PTpair units="PPCC" P= " 3.74E18" T= "219" precision="d"><me:bathGas>N2 </me:bathGas><me:experimentalRate ref1="HO2"ref2="HO2" refReaction="R1" error="1.83E-13">8.3E-13</me:experimentalRate></me:PTpair>
<me:PTpair units="PPCC" P= " 4.12E18" T= "219" precision="d"><me:bathGas>N2 </me:bathGas><me:experimentalRate ref1="HO2"ref2="HO2" refReaction="R1" error="1.647E-13">8.5E-13</me:experimentalRate></me:PTpair>
<me:PTpair units="PPCC" P= " 4.96E18" T= "219" precision="d"><me:bathGas>N2 </me:bathGas><me:experimentalRate ref1="HO2"ref2="HO2" refReaction="R1" error="1.83E-13">9.7E-13</me:experimentalRate></me:PTpair>

<!--Zabel   errors 0.9-->
<me:PTpair units="mbar" P= "1013" T= "260.95" precision="d"> <me:bathGas>N2</me:bathGas><me:experimentalRate ref1="HOONO2" ref2="HOONO2" refReaction="R1"error="8.64E-5">4.8e-4</me:experimentalRate></me:PTpair>
<me:PTpair units="mbar" P= "1015" T= "261.8" precision="d"> <me:bathGas>N2</me:bathGas><me:experimentalRate ref1="HOONO2"ref2="HOONO2" refReaction="R1" error="1.152E-4">6.4E-4 </me:experimentalRate></me:PTpair>
<me:PTpair units="mbar" P= "1014" T= "262.65" precision="d"><me:bathGas>N2</me:bathGas><me:experimentalRate ref1="HOONO2"ref2="HOONO2" refReaction="R1" error="9.9E-5">5.5e-4</me:experimentalRate></me:PTpair>
<me:PTpair units="mbar" P= "1011" T= "264.0" precision="d"> <me:bathGas>N2</me:bathGas><me:experimentalRate ref1="HOONO2"ref2="HOONO2" refReaction="R1" error="1.44E-4">8.2e-4</me:experimentalRate></me:PTp
<me:PTpair units="mbar" P= "1013" T= "264.2" precision="d"> <me:bathGas>N2</me:bathGas><me:experimentalRate ref1="HOONO2"ref2="HOONO2" refReaction="R1" error="1.53E-4">8.5e-4</me:experimentalRate></me:PTpair>
<me:PTpair units="mbar" P= "1013" T= "264.95" precision="d"> <me:bathGas>N2</me:bathGas><me:experimentalRate ref1="HOONO2" ref2="HOONO2" refReaction="R1"error="1.8E-4">10.0e-4</me:experimentalRate> </me:PTpair>
<me:PTpair units="mbar" P= "1027" T= "266.35" precision="d"> <me:bathGas>N2</me:bathGas><me:experimentalRate ref1="HOONO2"ref2="HOONO2" refReaction="R1"error=" 1.89E-4">10.9E-4 </me:experimentalRate></me:PTpair>
<me:PTpair units="mbar" P= "1011" T= "270.7" precision="d"> <me:bathGas>N2</me:bathGas><me:experimentalRate ref1="HOONO2"ref2="HOONO2" refReaction="R1" error="1.26E-4">23.2e-4</me:experimentalRate></me:PTpair>
<me:PTpair units="mbar" P= "1012" T= "270.7" precision="d"> <me:bathGas>N2</me:bathGas><me:experimentalRate ref1="HOONO2"ref2="HOONO2" refReaction="R1" error="3.96E-4">22.2e-4</me:experimentalRate></me:PTpair>
<me:PTpair units="mbar" P= "1010" T= "275.15" precision="d"><me:bathGas>N2</me:bathGas><me:experimentalRate ref1="HOONO2"ref2="HOONO2" refReaction="R1" error="8.1E-4">45.3e-4</me:experimentalRate></me:PTpair>

<me:PTpair units="mbar" P= "1012" T= "276.8" precision="d"> <me:bathGas>N2</me:bathGas><me:experimentalRateref1="HOONO2" ref2="HOONO2" refReaction="R1" error="9.9E-4">55.8e-4</me:experimentalRate>  </me:PTpair>
<me:PTpair units="mbar" P= "1013" T= "277.05" precision="d"> <me:bathGas>N2</me:bathGas><me:experimentalRate ref1="HOONO2" ref2="HOONO2" refReaction="R1" error="9.9E-4">56.6E-4 </me:experimentalRate> </me:PTpair>
<me:PTpair units="mbar" P= "1015" T= "277.1" precision="d"> <me:bathGas>N2</me:bathGas><me:experimentalRate ref1="HOONO2" ref2="HOONO2" refReaction="R1" error="9E-4">50.5e-4</me:experimentalRate>    </me:PTpair>
<me:PTpair units="mbar" P= "1014" T= "277.35" precision="d"> <me:bathGas>N2</me:bathGas><me:experimentalRate ref1="HOONO2" ref2="HOONO2" refReaction="R1" error="0.00108">56.94e-4</me:experimentalRate></me:PTpair>
<me:PTpair units="mbar" P= "1012" T= "278.85" precision="d"> <me:bathGas>N2</me:bathGas><me:experimentalRate ref1="HOONO2" ref2="HOONO2" refReaction="R1" error="0.00135">73.0e-4</me:experimentalRate></me:PTpair>
<me:PTpair units="mbar" P= "1015" T= "279.3" precision="d"> <me:bathGas>N2</me:bathGas><me:experimentalRateref1="HOONO2" ref2="HOONO2" refReaction="R1" error="0.00126">71.1e-4</me:experimentalRate></me:PTpair>
<me:PTpair units="mbar" P= "1010" T= "282.2" precision="d"> <me:bathGas>N2</me:bathGas><me:experimentalRate ref1="HOONO2" ref2="HOONO2" refReaction="R1" error="0.00189">103E-4 </me:experimentalRate></me:PTpair>
<me:PTpair units="mbar" P= "1017" T= "282.3" precision="d"> <me:bathGas>N2</me:bathGas><me:experimentalRate ref1="HOONO2" ref2="HOONO2" refReaction="R1" error="0.00198">109e-4</me:experimentalRate></me:PTpair>
<me:PTpair units="mbar" P= "1009" T= "282.4" precision="d"> <me:bathGas>N2</me:bathGas><me:experimentalRate ref1="HOONO2" ref2="HOONO2" refReaction="R1" error="0.00198">110e-4</me:experimentalRate></me:PTpair>
<me:PTpair units="mbar" P= "1013" T= "282.65" precision="d"> <me:bathGas>N2</me:bathGas><me:experimentalRate ref1="HOONO2" ref2="HOONO2" refReaction="R1" error="0.00216">121e-4</me:experimentalRate></me:PTpair>
<me:PTpair units="mbar" P= "1013" T= "282.9" precision="d"> <me:bathGas>N2</me:bathGas><me:experimentalRate ref1="HOONO2" ref2="HOONO2" refReaction="R1" error="0.00216">120e-4</me:experimentalRate></me:PTpair>
<me:PTpair units="mbar" P= "1013" T= "284.1" precision="d"> <me:bathGas>N2</me:bathGas><me:experimentalRate ref1="HOONO2" ref2="HOONO2" refReaction="R1" error="0.0027">149e-4</me:experimentalRate></me:PTpair>

<me:PTpair units="mbar" P= "212" T= "283.65" precision="d"> <me:bathGas>N2</me:bathGas> <me:experimentalRate ref1="HOONO2" ref2="HOONO2" refReaction="R1" error="7.92E-4">44.3e-4</me:experimentalRate></me:PTpair>
<me:PTpair units="mbar" P= "214" T= "282.95" precision="d"> <me:bathGas>N2</me:bathGas> <me:experimentalRate ref1="HOONO2" ref2="HOONO2" refReaction="R1" error="7.83E-4">43.4E-4 </me:experimentalRate></me:PTpair>
<me:PTpair units="mbar" P= "209" T= "283.05" precision="d"> <me:bathGas>N2</me:bathGas> <me:experimentalRate ref1="HOONO2" ref2="HOONO2" refReaction="R1" error="7.56E-4">42.1e-4</me:experimentalRate></me:PTpair>
<me:PTpair units="mbar" P= "212" T= "284.15" precision="d"> <me:bathGas>N2</me:bathGas> <me:experimentalRate ref1="HOONO2" ref2="HOONO2" refReaction="R1" error="9.54E-4">53.7E-4 </me:experimentalRate></me:PTpair>
<me:PTpair units="mbar" P= "55.4" T= "283.35" precision="d"> <me:bathGas>N2</me:bathGas> <me:experimentalRate ref1="HOONO2" ref2="HOONO2" refReaction="R1" error="3.06E-4">17.9e-4</me:experimentalRate></me:PTpair>
<me:PTpair units="mbar" P= "55.4" T= "283.4" precision="d"> <me:bathGas>N2</me:bathGas> <me:experimentalRate ref1="HOONO2" ref2="HOONO2" refReaction="R1" error="3.24E-4">18.5e-4</me:experimentalRate></me:PTpair>
<me:PTpair units="mbar" P= "56.1" T= "283.7" precision="d"> <me:bathGas>N2</me:bathGas> <me:experimentalRate ref1="HOONO2" ref2="HOONO2" refReaction="R1" error="3.366E-4">18.7e-4</me:experimentalRate></me:PTpair>
<me:PTpair units="mbar" P= "56.6" T= "283.7" precision="d"> <me:bathGas>N2</me:bathGas> <me:experimentalRate ref1="HOONO2" ref2="HOONO2" refReaction="R1" error="3.42E-4">19.5e-4</me:experimentalRate></me:PTpair>
<me:PTpair units="mbar" P= "14.1" T= "282.25" precision="d"> <me:bathGas>N2</me:bathGas> <me:experimentalRate ref1="HOONO2" ref2="HOONO2" refReaction="R1" error="9.9E-5">5.16E-4 </me:experimentalRate></me:PTpair>
<me:PTpair units="mbar" P= "14.1" T= "283.3" precision="d"> <me:bathGas>N2</me:bathGas> <me:experimentalRate ref1="HOONO2" ref2="HOONO2" refReaction="R1" error="1.08E-4">6.33e-4</me:experimentalRate></me:PTpair>
<me:PTpair units="mbar" P= "14.3" T= "283.35" precision="d"> <me:bathGas>N2</me:bathGas> <me:experimentalRate ref1="HOONO2" ref2="HOONO2" refReaction="R1" error="1.08E-4">5.91e-4</me:experimentalRate></me:PTpair>
<me:PTpair units="mbar" P= "15.2" T= "283.45" precision="d"> <me:bathGas>N2</me:bathGas> <me:experimentalRate ref1="HOONO2" ref2="HOONO2" refReaction="R1" error="1.17E-4">6.58e-4</me:experimentalRate></me:PTpair>
<me:PTpair units="mbar" P= "13.6" T= "289.35" precision="d"> <me:bathGas>N2</me:bathGas> <me:experimentalRate ref1="HOONO2" ref2="HOONO2" refReaction="R1" error="2.34E-4">13.8e-4</me:experimentalRate></me:PTpair>
<me:PTpair units="mbar" P= "14.0" T= "289.4" precision="d"> <me:bathGas>N2</me:bathGas> <me:experimentalRate ref1="HOONO2" ref2="HOONO2" refReaction="R1" error="2.16E-4">12.3e-4</me:experimentalRate></me:PTpair>
<me:PTpair units="mbar" P= "13.6" T= "298.05" precision="d"> <me:bathGas>N2</me:bathGas> <me:experimentalRate ref1="HOONO2" ref2="HOONO2" refReaction="R1" error="6.57E-4">36.9e-4</me:experimentalRate></me:PTpair>
<me:PTpair units="mbar" P= "13.6" T= "307.05" precision="d"> <me:bathGas>N2</me:bathGas> <me:experimentalRate ref1="HOONO2" ref2="HOONO2" refReaction="R1" error="0.00189">105e-4</me:experimentalRate></me:PTpair>
<me:PTpair units="mbar" P= "13.8" T= "307.25" precision="d"> <me:bathGas>N2</me:bathGas> <me:experimentalRate ref1="HOONO2" ref2="HOONO2" refReaction="R1" error="0.0018">102e-4</me:experimentalRate></me:PTpair>
<me:PTpair units="mbar" P= "13.6" T= "307.3" precision="d"> <me:bathGas>N2</me:bathGas> <me:experimentalRate ref1="HOONO2" ref2="HOONO2" refReaction="R1" error="0.0018">104e-4</me:experimentalRate></me:PTpair>

<!-- Ravi 40% error-->
<!-- <me:PTpair units="Torr" P= "52.1" T= "331.3" precision="d"> <me:bathGas>N2</me:bathGas> <me:experimentalRate ref1="HOONO2" ref2="HOONO2" refReaction="R1" error="0.084">0.21</me:experimentalRate></me:PTpair> -->
<!-- <me:PTpair units="Torr" P= "25.0" T= "331.7" precision="d"> <me:bathGas>N2</me:bathGas> <me:experimentalRate ref1="HOONO2" ref2="HOONO2" refReaction="R1" error="   0.136">0.34</me:experimentalRate></me:PTpair> -->
<!-- <me:PTpair units="Torr" P= "51.0" T= "334.4" precision="d"> <me:bathGas>N2</me:bathGas> <me:experimentalRate ref1="HOONO2" ref2="HOONO2" refReaction="R1" error="   0.148">0.37</me:experimentalRate></me:PTpair> -->
<!-- <me:PTpair units="Torr" P= "51.3" T= "337.9" precision="d"> <me:bathGas>N2</me:bathGas> <me:experimentalRate ref1="HOONO2" ref2="HOONO2" refReaction="R1" error="   0.292">0.73</me:experimentalRate></me:PTpair> -->
<!-- <me:PTpair units="Torr" P= "25.1" T= "341.6" precision="d"> <me:bathGas>N2</me:bathGas> <me:experimentalRate ref1="HOONO2" ref2="HOONO2" refReaction="R1" error="   0.424">1.06</me:experimentalRate></me:PTpair> -->
<!-- <me:PTpair units="Torr" P= "53.1" T= "342.4" precision="d"> <me:bathGas>N2</me:bathGas> <me:experimentalRate ref1="HOONO2" ref2="HOONO2" refReaction="R1" error="   0.748">1.87</me:experimentalRate></me:PTpair> -->
<!-- <me:PTpair units="Torr" P= "52.5" T= "343.2" precision="d"> <me:bathGas>N2</me:bathGas> <me:experimentalRate ref1="HOONO2" ref2="HOONO2" refReaction="R1" error="   0.440">1.1</me:experimentalRate></me:PTpair> -->
<!-- <me:PTpair units="Torr" P= "25.1" T= "347.3" precision="d"> <me:bathGas>N2</me:bathGas> <me:experimentalRate ref1="HOONO2" ref2="HOONO2" refReaction="R1" error="   0.588">1.47</me:experimentalRate></me:PTpair> -->
<!-- <me:PTpair units="Torr" P= "53" T= "349.9" precision="d"> <me:bathGas>N2</me:bathGas> <me:experimentalRate ref1="HOONO2" ref2="HOONO2" refReaction="R1" error="   1.272">3.18</me:experimentalRate></me:PTpair> -->

</me:PTs>

 </me:conditions>
  <me:modelParameters>
   <me:grainSize units="cm-1">100</me:grainSize>
    <me:energyAboveTheTopHill>25.0</me:energyAboveTheTopHill>
```

```
 </me:modelParameters>


<me:control>
  <me:calcMethod xsi:type="me:marquardt">
    <me:MarquardtIterations>15</me:MarquardtIterations>
    <me:MarquardtTolerance>1e-6</me:MarquardtTolerance>
    <me:MarquardtDerivDelta>0.025</me:MarquardtDerivDelta>
  </me:calcMethod>


  <me:testDOS />
  <me:printSpeciesProfile />
  <me:testMicroRates />
  <me:testRateConstant />
  <me:printGrainDOS />
  <me:printGrainkfE />
  <me:printGrainkbE />
  <me:eigenvalues>5</me:eigenvalues>
 </me:control>


<me:control>
  <me:calcMethod units="kJ/mol" xsi:type="me:ThermodynamicTable">
    <me:Tmin>0</me:Tmin>
    <me:Tmid>250</me:Tmid>
    <me:Tmax>500</me:Tmax>
    <me:Tstep>10</me:Tstep>
  </me:calcMethod>
</me:control>

  </me:mesmer>
```


References

1. Zabel, F., Unimolecular Decomposition of Peroxynitrates. *Zeitschrift Fur Physikalische Chemie-International Journal of Research in Physical Chemistry & Chemical Physics* **1995,** *188*, 119-142.

2. Gierczak, T.; Jimenez, E.; Riffault, V.; Burkholder, J. B.; Ravishankara, A. R., Thermal Decomposition of HO_2NO_2 (Peroxynitric Acid, PNA): Rate Coefficient and Determination of the Enthalpy of Formation. *J. Phys. Chem. A* **2005,** *109* (4), 586-596.

3. (a) Kurylo, M. J.; Ouellette, P. A., Rate Constants For the Reaction Ho2+No2+N-2-]Ho2no2+N-2 - the Temperature-Dependence of the Falloff Parameters. *Journal of Physical Chemistry* **1987,** *91* (12), 3365-3368; (b) Kurylo, M. J.; Ouellette, P. A., Rate Constants for the Reaction Hydroperoxy Radical + Nitrogen Dioxide + Nitrogen → Pernitric acid + Nitrogen: the Temperature Dependence of the Falloff Parameters. *J. Phys. Chem.* **1987,** *91* (12), 3365-3368; (c) Sander, S. P.; Peterson, M. E., Kinetics of the Reaction Perhydroxyl (HO_2) + Nitrogen Dioxide + M → Pernitric Acid (HO_2NO_2) + M. *J. Phys. Chem.* **1984,** *88* (8), 1566-1571.

4. Bacak, A.; Cooke, M. C.; Bardwell, M. W.; McGillen, M. R.; Archibald, A. T.; Huey, L. G.; Tanner, D.; Utembe, S. R.; Jenkin, M. E.; Derwent, R. G.; Shallcross, D. E.; Percival, C. J., Kinetics of the HO_2 + NO_2 Reaction: On the Impact of New Gas-phase Kinetic Data for the Formation of HO_2NO_2 on HOx, NOx and HO_2NO_2 Levels in the Troposphere. *Atmos. Environ.* **2011,** *45* (35), 6414-6422.

5. Troe, J.; Ushakov, V. G., Representation of "Broad" Falloff Curves for Dissociation and Recombination Reactions. *Z. Phys. Chem. (Muenchen, Ger.)* **2014,** *228* (1), 1-10.
